# Synthesis, Characterization, and Environmental Applications of Novel Per-Fluorinated Organic Polymers with Azo- and Azomethine-Based Linkers via Nucleophilic Aromatic Substitution

**DOI:** 10.3390/polym15204191

**Published:** 2023-10-23

**Authors:** Suha S. Altarawneh, Hani M. El-Kaderi, Alexander J. Richard, Osama M. Alakayleh, Ibtesam Y. Aljaafreh, Mansour H. Almatarneh, Taher S. Ababneh, Lo’ay A. Al-Momani, Rawan H. Aldalabeeh

**Affiliations:** 1 Department of Chemistry and Chemical Technology, Tafila Technical University, Tafila 66110, Jordan; osama.m.alakayleh@gmail.com (O.M.A.); ibtesam_aljaafreh@ttu.edu.jo (I.Y.A.); rawan.hanidalabeh@gmail.com (R.H.A.); 2 Department of Chemistry, Virginia Commonwealth University, Richmond, VA 23284, USA; helkaderi@vcu.edu (H.M.E.-K.); richardaj@vcu.edu (A.J.R.); 3 Department of Chemistry, University of Jordan, Amman 11942, Jordan; m.almatarneh@ju.edu.jo; 4 Department of Chemistry, Yarmouk University, Irbid 21163, Jordan; ababnehtaher@hotmail.com; 5 Department of Chemistry, Faculty of Science, The Hashemite University, Zarqa 13133, Jordan; loay.al-momni@hu.edu.jo

**Keywords:** per-fluorinated organic polymers, nucleophilic aromatic substitution, azomethine-based linkers, azo-based linkers, porosity measurements, environmental applications

## Abstract

This study reports on the synthesis and characterization of novel perfluorinated organic polymers with azo- and azomethine-based linkers using nucleophilic aromatic substitution. The polymers were synthesized via the incorporation of decafluorobiphenyl and hexafluorobenzene linkers with diphenols in the basic medium. The variation in the linkers allowed the synthesis of polymers with different fluorine and nitrogen contents. The rich fluorine polymers were slightly soluble in THF and have shown molecular weights ranging from 4886 to 11,948 g/mol. All polymers exhibit thermal stability in the range of 350–500 °C, which can be attributed to their structural geometry, elemental contents, branching, and cross-linking. For instance, the cross-linked polymers with high nitrogen content, DAB-Z-1h and DAB-Z-1O, are more stable than azomethine-based polymers. The cross-linking was characterized by porosity measurements. The azo-based polymer exhibited the highest surface area of 770 m^2^/g with a pore volume of 0.35 cm^3^/g, while the open-chain azomethine-based polymer revealed the lowest surface area of 285 m^2^/g with a pore volume of 0.0872 cm^3^/g. Porous structures with varied hydrophobicities were investigated as adsorbents for separating water-benzene and water-phenol mixtures and selectively binding methane/carbon dioxide gases from the air. The most hydrophobic polymers containing the decafluorbiphenyl linker were suitable for benzene separation, while the best methane uptake values were 6.14 and 3.46 mg/g for DAB-Z-1O and DAB-A-1O, respectively. On the other hand, DAB-Z-1h, with the highest surface area and being rich in nitrogen sites, has recorded the highest CO_2_ uptake at 298 K (17.25 mg/g).

## 1. Introduction

Perfluorinated organic polymers (PFPs) with extended ether bonds are a widely recognized type of multi-functional, open-chain organic polymers used in the fields of medicine, biomedical research, electronics, optoelectronics, high-quality coatings, sensors, and environmental applications [1,2,3,4,5]. They are synthesized by introducing fluorinated linkers into the polymer chains [6]. The C-F bond exhibits strong polarization and has a high bond energy (approximately 480 kJ/mol), which makes PFPs suitable for various usages [1]. The synthesis of PFPs has been utilized in the polymer industry to produce novel materials with several desirable properties, such as high-temperature stability, high glass transition temperature, resistance to solvents, and applicability for gas separation. PFPs also exhibit flame retardancy, which makes them useful in various implementations [7,8]. PFPs possess tunable properties such as thermal and oxidative stability, hydrophobicity, lipophobicity, dielectric properties, and adjustable polarity. This enables their utilization in a diverse array of fields [9,10].

PFPs have been utilized in the synthetic modification of insoluble organic polymers by incorporating perfluorinated chains into their frameworks. This approach has improved the processability (e.g., solubility) of various insoluble organic polymers, such as poly(chains), ether, imine, azo-based, amide, ketone, and sulfone, in non-polar media. This improvement can be achieved by introducing fluorinated linkers known for their hydrophobic nature (immiscibility in water) and very low dipole [3,4,9,11]. In addition, to their enhanced processability, the catalytic reduction reactions of imine, amide, or carbonyl groups within the polymer chains would open new channels for their usage [12]. 

Furthermore, the presence of fluorinated chains within the polymer framework has been employed to enhance the casting and moldability of various polyethers. For instance, NORYL™, a popular polyether polymer composed of polyphenylene oxide, features a high aromatic content and polymer aggregation chains that restrict its solubility. To improve the moldability of NORYL™ for numerous applications, plasticizing additives such as polystyrene have been compounded with it [13]. Nevertheless, improving the processability of the polymer is often accompanied by a decrease in its thermal stability and resistance to oxidation [14]. Thus, PPO was modified by selectively introducing hexafluorobenzene, decafluorobiphenyl, and bisphenol AF into its polymer backbone to enrich the chains with fluorine substituents, thereby enhancing its solubility and thermal stability.

Recent developments in PFPs have focused on the class of fluorinated ether-based polymers [3]. These polymers have been synthesized through metal-catalyzed coupling reactions and nucleophilic aromatic substitution (NAS) reactions between diols and fluorinated linkers [15,16,17]. In the NAS synthetic methodology, successful substitution of the C-F bond is achieved by using strong nucleophiles containing oxygen, such as aromatic phenoxide or aliphatic alkoxides. This methodology is employed with various small molecules or polymer chains such as fluorinated poly(aryl ether), poly(aliphatic ethers), poly(ether ketone)s, poly(ether sulfone)s, and poly(ether nitrile)s [11,18,19,20,21,22]. The above-mentioned principle is also employed in the preparation of fluorinated polyamines or fluorinated poly(sulfide) using diamines and dithiols [23,24]. Furthermore, hybrid chains have been reported in the literature that demonstrate the presence of conjugated moieties composed of poly(aryl ether)s alternating with poly(imide)s, poly(azomethine), or poly(diazo-based) units [3,20,25,26].

It is worth noting that the synthesis of para-connected, open-chain fluorinated polymers with optimal solubility was achieved using mild polymerization conditions. This involved the use of a weak base with an optimized monomer ratio, a low feeding order, and low reaction temperatures [26]. Nonetheless, these mild polymerization conditions are associated with low formation yields and long polymerization times of the polymers [14]. In contrast, utilizing harsher polymerization conditions results in optimal yields and time while producing randomly agglomerated frameworks with high cross-linking rates and minimal solubility. These cross-linked frameworks are created due to the rapid rate of NAS at high temperatures, leading to the substitution of C-F bonds at both para- and ortho-positions of the aromatic linkers, which assisted in the formation of randomly branched chains [27].

Most of the existing literature has primarily focused on the synthesis of perfluorinated organic polymers as open chains and their potential applications, with limited studies on the advantageous formation of cross-linked fluorinated polymers. However, recent work has reported the development of new cross-linked fluorinated poly(aryl ether) (C-FPAE) films. These films exhibit excellent thermal stability up to 495 °C, dimensional stability, hydrophobic properties, and a high storage modulus in high-temperature environments. Additionally, they serve as a low dielectric material [17]. Another recent study reported the synthesis of new cross-linked fluorinated polymers that functioned as a supporting skeleton for controlling the structural integrity of Al/oxidizer microspheres in the production process of composite propellants [28].

After conducting a thorough review of the literature, we have observed a limited number of reports emphasizing the potential advantages of synthesizing fluorinated cross-linked frameworks. Furthermore, there is a lack of research on the opportunity to create high-surface-area and porous frameworks through cross-linking, which would enhance their potential for environmental applications such as separation.

The absence of sufficient research in this area has motivated us to focus on synthesizing a new series of fluorinated organic polymers and investigating the factors that impact their solubility, polarity, and porosity after cross-linking. Our initial phase involves incorporating diol-based linkers, specifically nitrogen-rich organic linkers such as azomethine and azo-based compounds, connected with fluorinated linkers into the new polymers. These combinations were specifically chosen to study the impact of fluorine content on the solubility of poly(azomethine)s and poly(diazo-based) polymers. Additionally, we investigated the influence of factors such as the size of the fluorinated linker, the connection site (ortho or para), and the cross-linking polymerization conditions. The newly synthesized polymers were tested as solid sorbents for phenol, benzene, carbon dioxide, and methane. The confirmation of successful polymer preparation through the NAS reaction was achieved using the ^19^F NMR technique. Additionally, we utilized various characterization techniques to verify the synthesis of the polymers and establish their porosity. This study also presents the environmental applications of these polymers in separating the selected adsorbates.

## 2. Material and Methods

### 2.1. Materials

All chemicals used in this study were purchased from Sigma-Aldrich (St. Louis, MO, USA) and used without additional purification, except where specified. The polymer synthesis was conducted using the Schlenk line technique in the presence of a Dean-Stark trap glassware equipment, and under a nitrogen atmosphere. The structures of the linkers and the polymers were confirmed using ^1^H NMR and ^13^C NMR techniques. Meanwhile, ^19^F NMR was only utilized for the analysis of polymers. These measurements were conducted using a Bruker 500 MHz Avance III spectrometer (Karlsruhe, Germany) in deuterated DMSO (DMSO-d_6_ at 298 K). The FTIR spectra of all structures were recorded using a Shimadzu FTIR-8300 (Long Beach, CA, USA), employing KBr pellets in the range of 4000–600 cm^−1^. Thermogravimetric analysis (TGA) of the polymers was performed using a Perkin Elmer TGA 800 device. The samples were heated under air from 30 to 850 °C, with a heating rate of 10 °C/min. Molecular weight measurements were carried out by gel permeation chromatography (GPC) using a Waters 510 system equipped with a UV detector set at 254 nm. THF was used as an eluent, and the calibration was made with polystyrene standards. The Brunauer–Emmett–Teller (BET) surface area and methane and carbon dioxide uptakes at 298 K were obtained using a TriStar II 3020 surface area and porosity analyzer (Micromeritics, Norcross, GA, USA). The surface area was measured using argon gas at 87 K and used to determine the pore size distribution. Prior to conducting the porosity measurements, the samples were soaked in methanol for 12 h and degassed under vacuum at a temperature of 150 °C for a duration of 12 h.

### 2.2. Synthesis of Organic Linkers

#### 2.2.1. Synthesis of 1,3-(1-azomethine-2-hydroxyphenyl)benzene (DAB-A-OH)

DAB-A-OH was synthesized using a Schiff-base condensation reaction, following the procedures described in previous literature [29]. In the synthetic protocol, a solution of aromatic diamine (2.0 mmol) in 10.0 mL of absolute ethanol was added gradually over a 15 min period to a solution containing salicylaldehyde (4.0 mmol) dissolved in 10.0 mL of absolute ethanol. A catalytic amount of glacial acetic acid (1.0 mL) was then added to the solution, resulting in a yellow-colored mixture. The solution was then refluxed for 6 h. The resulting precipitate was filtered, washed multiple times using water and diethyl ether, and dried under a vacuum. The final product was a yellow solid that showed solubility in DMF, DMSO, and CHCl_3_. The obtained yield was 87%, and the melting point was recorded to be between 127 and 129 °C. ^1^H NMR (500 MHz, DMSO-d_6_): δ (ppm) = 6.98 (s, 1H, OH), 7.02–7.69 (m, 11H, Ar-H), 9.06 (s, 2H, HC=N-), and 13.04 (s, 2H, Ar-OH). ^13^C NMR (500 MHz, DMSO-d_6_): δ (ppm) = 114.33, 117.11, 119.72, 120.66, 130.83, 133.11, 133.94 (Ar-C), 149.73 (N-C-Ar), 160.79 (-C=N-), and 164.64 (ArC-OH) (Note: Ar = Aromatic) (Figures S1 and S2).

#### 2.2.2. Synthesis of 1,3-(1-azo-4-hydroxyphenyl)benzene (DAB-Z-OH)

The synthesis of DAB-Z-OH involved a diazotization reaction, as per previously reported literature [30]. To synthesize DAB-Z-OH, two separate solutions were prepared as solutions 1 and 2, and then the solutions were mixed by drop-wise addition. Solution 1 was prepared by dissolving 1,3-diaminobenzene (DAB) (3.0 mmol) in 30 mL of distilled water, and then the solution was stirred for 15 min. After that, concentrated hydrochloric acid (9 mmol) was added dropwise while maintaining a temperature of 0–4 °C. Solution 2 was prepared by dissolving sodium nitrite (6 mmol) in 10 mL of distilled water, then added dropwise to Solution 1. Once the diazotization process was complete, the mixture was stirred for an hour while the temperature was kept around 4 °C. The solution was slowly added to a mixture of 6 mmol of phenol, 10 mL of ethanol, and a buffer solution containing acetic acid and sodium acetate with a pH of 6.5. The final mixture was then stirred in an ice bath for two hours to produce the dye. The prepared dye was filtered, washed with cold water, and dried under a vacuum. The final product was a red crystalline solid soluble in methanol, DMSO, and DMF. The yield of the product was 85%, and the melting point was below 300 °C, beyond which it decomposed. ^1^H NMR (500 MHz, DMSO-d_6_): δ (ppm) = δ 6.92 (d, 4H, Ar-OH), 7.73 (d, 4H, Ar-OH), 7.74 (q, 1H, Ar-H), 7.79 (d, 2H, Ar-H), 8.16 (s, 1H, Ar-H), and 10.43 (s, 2H, Ar-OH). ^13^C NMR (500 MHz, DMSO-d_6_): δ (ppm) = 115.99, 124.55, 125.12, 130.27, 145.11 (Ar-C), 161.32 (ArC-OH), and 152.86 (C-N=N-) (Figures S3 and S4).

### 2.3. General Synthesis of Per-Fluorinated Polymers

The synthesis of the new polymers was depicted as follows: diphenol linkers (0.44 mmol) of either DAB-A-OH or DAB-Z-OH and potassium carbonate (0.52 mmol) were dissolved in 10 mL of toluene and 50 mL of dimethylacetamide (DMAc) in a 250 mL Schlenk flask. The reaction flask was connected to a Dean-Stark trap, and the solution refluxed for 6 h and was purged with an inert nitrogen flow to create an oxygen-free environment. The excess toluene was then distilled off to produce the phenoxide anion. The solution was then cooled and added gradually to a solution of hexafluorobenzene (HFB) or decafluorobiphenyl (DFB) (0.44 mmol) dissolved in DMAc. The resulting mixture was then stirred for three hours at room temperature and heated to 60 °C for 48 h. After that, the mixture was poured into a 1000 mL beaker that contained a concentrated mixture of HCl, a slurry of ice, and methanol (0.5:1:1). The formed precipitate was then filtered, washed with water, and dried under a vacuum. The final products were pale to dark brown and showed slight solubility in THF, diethyl ether, DMSO, and CHCl_3_. The physical and spectral data of the product are as follows:

#### 2.3.1. Poly(4,4-(diazomethine phenyl)-o-diphenoxy-tetrafluorbenzene) (DAB-A-1h)

**Figure 1:**
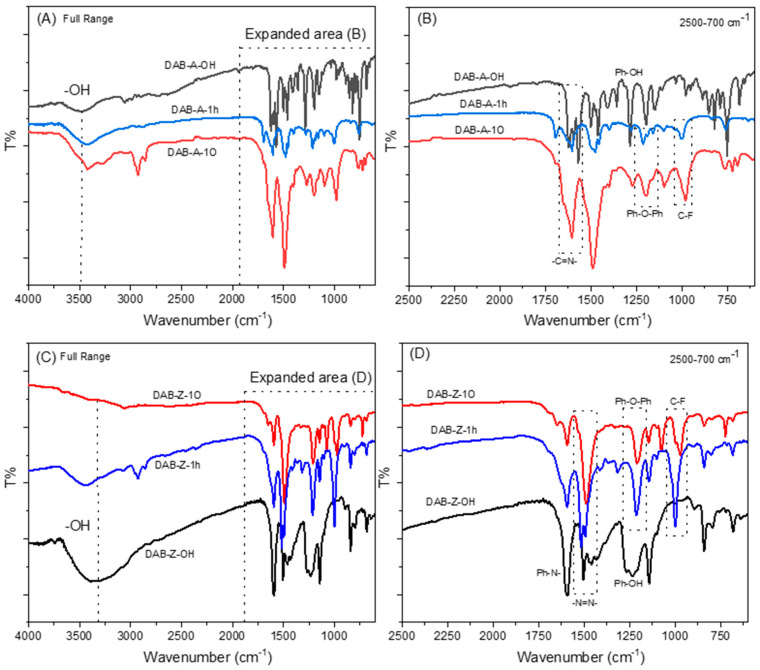
FTIR in the full and expanded ranges: (**A**,**B**) DAB-A-OH and the corresponding polymers; (**C**,**D**) DAB-Z-OH and the corresponding polymers.

**Figure 2:**
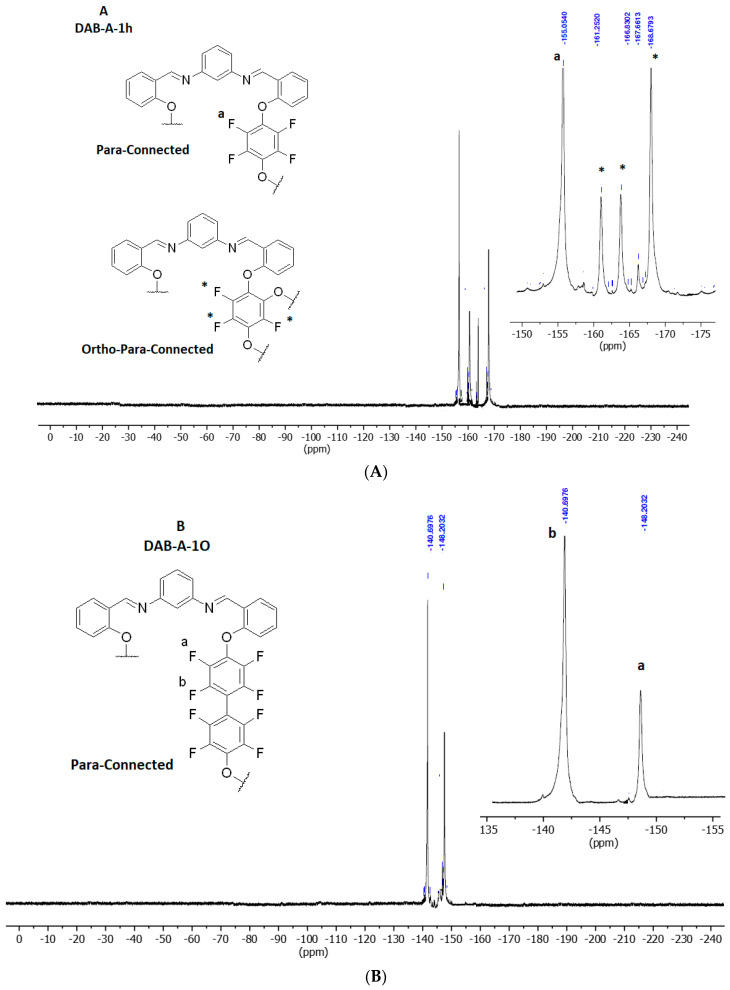
(**A**) ^19^F NMR spectra of the DAB-A-1h in DMSO. (**B**) ^19^F NMR spectra of DAB-A-1O in DMSO. (*: Peaks related to the polymerization on the ortho-sides).

DAB-A-1h was synthesized by the polymerization of DAB-A-OH and HFB, resulting in a brown solid with a yield of 0.22 g, 82%); FTIR (KBr) ν cm^−1^: 3600–3000 (Ar-OH), 2800–3000 Ar(-C-H), 1600 (-C=N-), 1475–1505 Ar(-C=C-), 1216 Ar(C-O-C), 1007 (C-F) (Figure 1A,B). ^1^H NMR (500 MHz, DMSO-d_6_): δ (ppm) = Ar-H (7.21- 7.82), weak broad signals (9.67, 10.49), aysmmetirc (2H of H-C=N-). ^13^C NMR (500 MHz, DMSO-d_6_): δ (ppm) = Ar-C (115.49–130.9), C-F (139.42, 141.05, 140.94, 144.0), (-C=N) 170.17, Ar(C-N) 156.04, and Ar(C-O) 158.71 ppm. ^1^H NMR and ^13^C NMR are shown in Figures S5 and S6. ^19^F NMR (500 MHz, DMSO-d_6_): δ (ppm) appeared at −154.66, corresponding to the para-fluorine of HFB after polyermization. The δ (ppm) at −155.05, −157.89, −157.93, −160.81, −161.25, −166.77, −167.66, and −168.67 are related to the random polymerization (on ortho and para) of HFB (Figure 2A).

#### 2.3.2. Poly(4,4-(diazomethine phenyl)-o-diphenoxy-octafluorobiphenyl) (DAB-A-1O)

DAB-A-1O was produced by polymerizing DAB-A-OH and DFB, resulting in a brown and solid polymer with a weight of 0.20 g and a yield of 69%. The FTIR spectrum (KBr) revealed peaks at 3600–3000 cm^−1^ (Ar-OH), 2800–3000 cm^−1^ (Ar(-C-H)), 1608 cm^−1^ (-C=N-), 1488–1505 cm^−1^ Ar(-C=C-), 1194 cm^−1^ Ar(C-O-C), and 979 cm^−1^ (C-F) (Figure 1A,B). ^1^H NMR (500 MHz, DMSO-d_6_): δ (ppm) = Ar-H (6.51–8.63) appeared as weak broad signals. ^13^C NMR (500 MHz, DMSO-d_6_): δ (ppm) = Ar-C (119.66–129.68), C-F (136.73–145.80), (-C=N) 170.03, Ar(C-N) 162.24, and Ar(C-O) at 160.0 ppm. Both the ^1^H NMR and ^13^C NMR spectra are included in Figures S7 and S8. The ^19^F NMR (500 MHz, DMSO-d_6_): δ (ppm) = −140.69 and −148.2, indicating para-polymerization of DFB with no further random polymerization peaks (Figure 2B).

#### 2.3.3. Poly(4,4-(diazophenyl)-p-diphenoxy-tetrafluorbenzene) (DAB-Z-1h)

**Figure 3:**
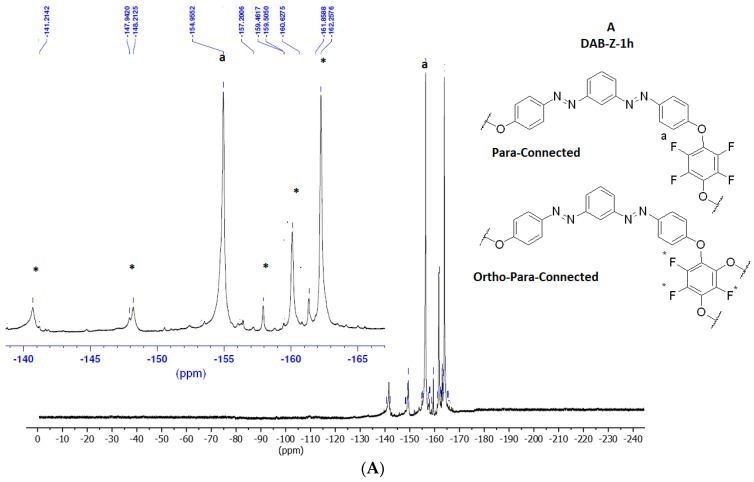

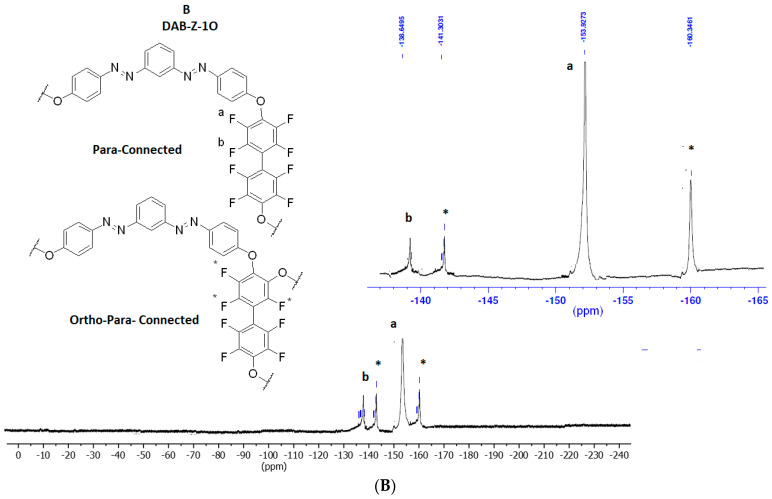
(**A**) ^19^F NMR spectra of DAB-Z-1h in DMSO. (**B**) ^19^F NMR spectra of DAB-Z-1O in DMSO. (*: Peaks related to the polymerization on the ortho-sides).

DAB-Z-1h was obtained by polymerizing DAB-Z-OH and HFB, resulting in a brown and solid polymer with a weight of 0.22 g and a yield of 88%. The FTIR spectrum (KBr) showed peaks at 3600–3000 cm^−1^ (Ar-OH), 2800–3000 cm^−1^ (Ar(-C-H)), 1518 cm^−1^ (-N=N-), 1500–1560 cm^−1^ (Ar(-C=C-)), 1211 cm^−1^ (Ar(C-O-C)), and 997 cm^−1^ (C-F) (Figure 1C,D). ^1^H NMR (500 MHz, DMSO-d_6_): δ (ppm) = Ar-H (6.51–8.63), appeared as weak broad signals. ^13^C NMR (500 MHz, DMSO-d_6_): δ (ppm) = Ar-C (116.15–116.94, 122.88–137.44), Ar(C-O-C) 170.0, and (C-N=N-) 155.0 and 160.0, C-F appeared (139.42, 140.05, 140.94, 143.00, 143.75, 148.60, 153.0) ppm. Both ^1^H NMR and ^13^C NMR are displayed in Figures S9 and S10. ^19^F NMR (500 MHz, DMSO-d_6_): δ (ppm) = −154.95 corresponding to the polymerization at the para-positions of HFB, while the peaks at −140.70, −147.97, −148.21, −157.93, −160.62, −161.35, and −162.25 were related to further random connections at the ortho- and para-positions of HFB (Figure 3A).

#### 2.3.4. Poly(4,4-(diazophenyl)-p-diphenoxy-octafluorobiphenyl) (DAB-Z-1O)

DAB-Z-1O was synthesized by polymerizing DAB-Z-OH and DFB, resulting in a brown and solid polymer with a weight of 0.26 g and a yield of 70%. The FTIR spectrum (KBr) displayed peaks at 3600–3000 cm^−1^ (Ar-OH), 2800–3000 cm^−1^ (Ar(-C-H)), 1491 cm^−1^ (-N=N-), 1500–1560 cm^−1^ (Ar(-C=C-)), 1211 cm^−1^ (Ar(C-O-C)), and 972 cm^−1^ (C-F) (Figure 1C,D). ^1^H NMR (500 MHz, DMSO-d_6_): δ (ppm) = Ar-H (7.43–8.85) appeared as weak broad signals. ^13^C NMR (500 MHz, DMSO-d_6_): δ (ppm) = Ar-C (119.9–129.0), (C-F) (143.42, 144.05, 144.94, 146.00), Ar(C-O-C) 180.0), and (C-N=N-) at 170.0 and 155.0 ppm. Both ^1^H NMR and ^13^C NMR are shown in Figures S11 and S12. ^19^F NMR (500 MHz, DMSO-d_6_): δ (ppm) = −138.88 and −153.97 related to the polymerization on the para-position of DFB, while the peaks at −138.64, −141.30, −160.34, and −160.38 were attributed to random polymerization (Figure 3B).

## 3. Results and Discussion

### 3.1. Synthesis of Organic Linkers

**Scheme 1:**
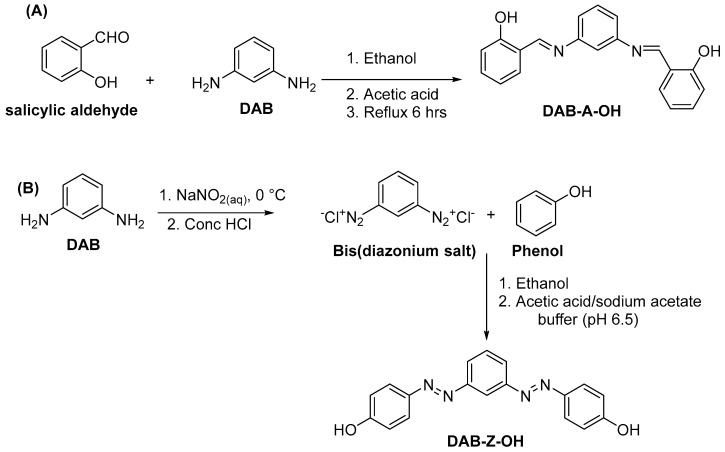
Synthetic routes of linkers (**A**) DAB-A-OH and (**B**) DAB-Z-OH (DAB: 1,3-diaminobenzene).

The aim of this study is to synthesize a new series of open-chain fluorinated organic polymers via a nucleophilic aromatic substitution (NAS) reaction. This type of polymer can be prepared through the polymerization of di-phenols and aromatic fluorinated linkers [5,15]. In this work, the polymers were prepared via the NAS of diphenols, DAB-A-OH and DAB-Z-OH, with HFB and DFB. DAB-A-OH and DAB-Z-OH correspond to azomethine-based and diazo-based, respectively. DAB-A-OH was obtained through the condensation reaction of salicylaldehyde with 1,3-diaminobenzene (DAB) under reflux conditions for 24 h in an acid-catalyzed medium (acetic acid), as shown in Scheme 1A. The final product was a yellow solid with good solubility in various solvents such as DMSO, DMAc, DMF, methanol, and ethanol. The authenticity of the product was confirmed through ^1^H, ^13^C NMR, and FTIR analysis. Confirmation of the linker formation was achieved through ^1^H NMR analysis, where the proton of an imine bond (-HC=N-) was observed at δ 9.06, and the incorporation of the phenol group (proton of OH) was seen at δ 13.04, as demonstrated in Figure S1. Similarly, ^13^C NMR analysis of DAB-A-OH displayed distinct carbon regions (Figure S2), with two prominent carbons of the imine bond (-C=N-) and (C-OH) observed at δ 160.79 and 164.64, respectively. The FTIR spectrum within the range of 4000–600 cm^−1^ and the expanded area of 2500–700 cm^−1^ (Figure 1A,B) allowed for the identification of the hydroxyl of Ar-OH and the imine bond (-C=N-) at 3600–3000 and 1622 cm^−1^, respectively.

The diazo-based linker DAB-Z-OH was synthesized through the diazotization reaction of the appropriate 1,3-diaminobenzene (DAB), followed by coupling of the bis(diazonium salt) with di-phenol, as shown in Scheme 1B. The final product obtained was a dark brown solid, soluble in polar solvents such as DMAc and DMSO. The ^1^H NMR revealed all protons appearing in the aromatic region, while the OH- group proton was determined at δ 10.43. Notably, the prominent carbons related to phenol-C and (C-N=N-) were observed at δ 161.3 and 152.9, respectively (Figures S3 and S4). The FTIR spectrum covering the full range of 4000–600 cm^−1^ and the expanded area of 2500–700 cm^−1^ (Figure 2A,B) allowed for the identification of bands corresponding to (Ar-OH), Ar(-C-N), and (-N=N-) at 3600–3000, 1590, and 1500 cm^−1^, respectively.

### 3.2. Synthesis of Perfluorinated Polymers

**Scheme 2:**
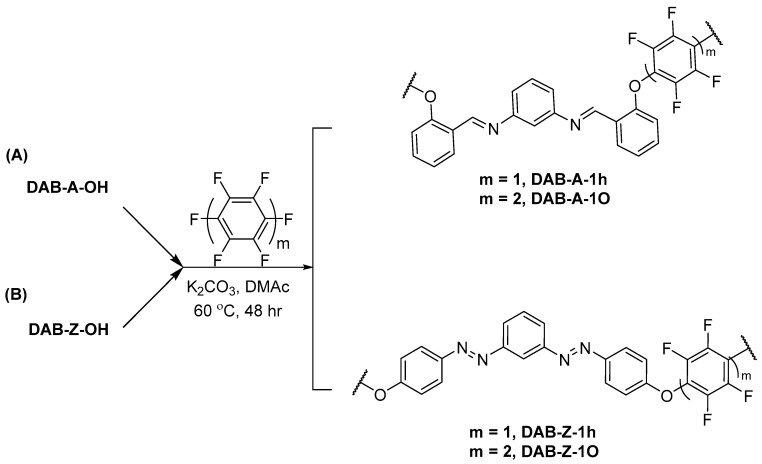
Synthesis protocol of (**A**) DAB-A-1O and DAB-A-1h; (**B**) DAB-Z-1O and DAB-Z-1h (m = the number of fluorinated phenyls; the present structure of each polymer represents one repeating unit).

Following the characterization of the di-phenol organic linkers, we have synthesized four new fluorinated polymers by coupling (1:2.5 mmol) of the linkers DAB-A-OH and DAB-Z-OH with HFB and DFB, respectively, as illustrated in Scheme 2. The newly produced series of polymers were named DAB-A-1h, DAB-A-1O, DAB-Z-1h, and DAB-Z-1O, where the symbols (h) and (O) signify hexa- and octa-groups, respectively, and (A) and (Z) correspond to azomethine and azo-based groups, respectively. The polymers were synthesized using a one-step polycondensation reaction between di-phenols and fluorinated linkers in the presence of potassium carbonate as a base through the nucleophilic aromatic substitution (NAS) reaction. The formation of the bis-phenoxide basic sites was followed by a nucleophilic substitution reaction at the C-F bond, resulting in the anticipated extended ether (C-O-C) chains. The general mechanism for this reaction is depicted in Scheme S1.

As outlined in the synthetic methodology, the polymers were precipitated in an acidic solution, filtered, and washed with water several times to eliminate any residual potassium fluoride salt. The final products obtained were pale to dark brown solids with good yields (Table 1). While all the polymers exhibited slight solubility in DMSO, DMAc, and THF, the polymer DAB-A-1O was completely soluble in diethyl ether and THF, as demonstrated in Figure S13. The slight to good solubility of the polymers in THF has allowed us to determine their molecular weight by applying gel permeation chromatography (GPC) measurements. As summarized in Table 1, DAB-A-1O is soluble in THF and has shown the highest number and weight average molecular weights (Mn = 4068, Mwt = 11,948 g/mol). The other polymers have shown lower values due to their slight solubility during the measurements. DAB-Z-1h is insoluble in THF, so we could not apply the measurement. Confirmation of the formation of ether bonds in all the polymers was achieved through ^1^H and ^13^C NMR analysis, as shown in Figures S5–S12.

The ^1^H NMR spectra of DAB-A-1h and DAB-A-1O have shown the disappearance of the terminal hydroxyl group (OH) proton, which appears around 13 ppm in the spectrum of the linker DAB-A-OH (Figures S5 and S7). Similarly, in the polymers DAB-Z-1h and DAB-Z-1O, the disappearance of the terminal hydroxyl group proton, located around 11 ppm, confirmed the polymerization (Figures S9 and S11). The ^13^C NMR spectra of the polymers validated the integration of the fluoro-aromatic linkers, evident from the C-F chemical shifts appearing within the range of 137–144 ppm. Additionally, the disappearance of the OH group and the subsequent formation of the C-O-C ether linker occurred around 170 ppm, while the inclusion of phenyl-N and C=N was observed at 155–159 ppm (Figures S6 and S8). In contrast, the presence of phenyl-N=N was confirmed at 155–160 ppm in the ^13^C NMR spectra of polymers DAB-Z-1h and DAB-Z-1O (Figures S10 and S12). The limited resolution of the peaks observed in the ^1^H and ^13^C NMR spectra of the polymers may be attributed to the slight solubility of the polymers in the polar solvent DMSO. Moreover, the cross-linking of the polymer chains could have contributed to the enhanced aggregation, further impacting the resolution of the peaks.

The FTIR spectra of the polymers in the range of 4000–600 cm^−1^, along with the expanded area of 2500–700 cm^−1^, demonstrated significant changes due to the incorporation of fluorinated benzene HFB and DFB in the polymer chains. Notably, the broad area of the OH group around 3000 cm^−1^ gradually decreased upon polymerization, and the observed broadening may be attributed to the presence of unpolymerized terminals of the linkers DAB-Z-OH or DAB-A-OH. Confirmation of the successful NAS reaction at the fluoro-sites was established through the formation of the C-O-C bond around 1250 cm^−1^ and the integration of the C-F bond around 1000 cm^−1^. The inclusion of the diazo (N=N) and azomethine (C=N) groups was evident in the range of 2500–500 cm^−1^, appearing at 1500 and 1630 cm^−1^, respectively. The FTIR spectra of the polymers, along with their corresponding monomers, are presented within the range of 4000–600 cm^−1^ and the expanded area of 2500–700 cm^−1^, as demonstrated in Figure 1.

The morphology of the polymers was determined by scanning electron microscopy (SEM). The polymers have irregularly agglomerated particles, as shown in Figure S14. 

### 3.3. Nucleophilic Aromatic Substitution (NAS) and the Cross-Linking Process

As mentioned earlier, the synthesis of novel ether-linked perfluorinated polymers was achieved through the NAS mechanism [31]. The polymerization process involved the deprotonation of DAB-A-OH and DAB-Z-OH to generate bis-phenoxide anion (Nu:), followed by the nucleophilic attack of the fluorine positions of the fluorinated linkers such as HFB and DFB. The nucleophilic attack occurred at the (C-F) bond, which was ortho or para to another C-F bond, thereby facilitating the substitution reaction. The detailed steps of the reaction are presented in Scheme S1. The polymerization conditions employed in this study have been utilized in the preparation of numerous previously reported perfluorinated organic polymers [1,3,10,23]. As stated in the literature, the preparation of polymers containing HFB or DFB linkers was conducted under mild conditions, with relatively low temperatures not exceeding 80 °C, along with a prolonged stirring time [25]. In this instance, the polymers were created as para-connected extended open chains that exhibited low yield while displaying good solubility in non-polar solvents [32,33]. In contrast, performing the polymerization at high temperatures resulted in the formation of polymers with good yield and limited solubility. For example, a series of perfluorocyclobutyl-based polymers were synthesized at polymerization temperatures ranging from 160–200 °C. These polymers exhibited limited solubility in most organic solvents and formed random-agglomerated chains, referred to as cross-linked frameworks [34]. In this regard, we have focused on obtaining extended cross-linked chains that exhibit a porosity nature. To reach this target, we have performed the experimental protocol under optimal conditions where the temperature did not exceed 60 °C with gradual heating over 48 h. 

To identify the possibility of forming open-chain or cross-linked polymers, we conducted an analysis of the polymers using ^19^F NMR. Given that the polymerization temperature reached 60 °C, random NAS and the formation of agglomerated chains were anticipated. The ^19^F NMR spectra of the polymers were acquired using DMSO-d_6_ solvent, and the chemical shifts of the fluorine groups were determined and compared with J-coupled spectroscopy (JCS) calculations (Table S1) [35]. In this study, we investigated the ^19^F NMR spectra of the new polymers and proposed their formation based on the following two aspects:

#### 3.3.1. Azomethine-Based Fluorinated Polymers

The azomethine-based polymers (DAB-A-1h and DAB-A-1O) were synthesized through the polymerization of HFB or DFB with DAB-A-OH, respectively (Scheme 2). According to the NAS, the anticipated structure of DAB-A-1h should exhibit one type of ^19^F located at the −155 ppm position, which corresponds to the fluorine of the C-F bonds labeled (a) in Figure 2A. However, the ^19^F NMR spectrum of DAB-A-1h exhibited three additional peaks at positions −157.8, −160.8, and −167 ppm, which can be attributed to the formation of side products resulting from the polymerization on the ortho-sides labeled (*). In contrast, the ^19^F NMR spectrum of DAB-A-1O confirmed the successful and controlled para-substituted open chain through the presence of two distinctive peaks at −141 and −148 ppm, as shown in Figure 2B. These peaks were assigned to the two different types of fluorine in the C-F bonds on the biphenyl linker, labeled (a) and (b) in Figure 2B.

The formation of the polymers can also be influenced by the size and type of linkers used, in addition to the polymerization conditions. In this case, both DFB and HFB were linked to the phenoxide group, which was located ortho to the -C=N- group. The steric factor that resulted from connecting DFB and HFB to the phenoxide group, which was ortho to the -C=N- group, played a role in the formation of the polymers. This connection contributed to further structural collapse. Due to the steric effect and the large size of DFB, the accessible sites for further NAS were limited, resulting in an open-chain polymer (DAB-A-1O). On the other hand, even though DAB-A-1h was synthesized under the same conditions, the small size of HFB facilitated random NAS. The higher reactivity of HFB was due to the flexible rotation of the linker, which provided more available fluorine sites for nucleophilic substitution during the polymerization process. DAB-A-1O, which had a larger DFB linker, had limited sites for further NAS, leading to the formation of an open-chain polymer. DAB-A-1O had good solubility in non-polar solvents, such as THF and diethyl ether, owing to its hydrophobic nature and high fluorine content (Figure S13). On the other hand, the pronounced solubility of DAB-A-1h and DAB-A-1O in polar solvents, such as DMAc, DMSO, and chloroform, was attributed to the presence of the nitrogen atom in the -C=N group [9,11].

#### 3.3.2. Diazo-Based Fluorinated Polymers

Fluorinated polymers with diazo-based linkers (DAB-Z-1h and DAB-Z-1O) were prepared by coupling DAB-Z-OH with HFB and DFB, respectively. The ^19^F NMR spectra of both polymers indicated the formation of cross-linked structures in addition to the expected open chains. The linker DAB-Z-OH contained hydroxyl groups adjacent to the azo group, unlike DAB-A-OH (Scheme 1B). The presence of hydroxyl groups adjacent to the azo group in DAB-Z-OH increased the accessibility of the fluorine atoms for the NAS during polymerization. The free rotation of the fluorinated linkers allowed for random substitution of the phenoxide on the C-F sites, which led to the elimination of steric factors and facilitated the NAS. As a result, cross-linking occurred, forming both expected open chains and cross-linked structures in DAB-Z-1h and DAB-Z-1O, as confirmed by their ^19^F NMR spectra. The ^19^F NMR spectrum of DAB-Z-1h indicated a peak at −154.95 ppm labeled (a) in Figure 3A, which was assigned to the fluorine of the C-F bonds of the para-connected polymer. However, the other peaks at −140.70, −147.97, −148.21, −157.93, −160.62, −161.35, and −162.25 ppm were attributed to C-F bonds that were formed due to the random polymerization, as shown in Figure 3A. Similarly, in the ^19^F NMR spectrum of DAB-Z-1O, two peaks at −138.88 and −153.97 ppm were observed, labeled (a) and (b) in Figure 3B, corresponding to the C-F bonds of para-connected chains. Other peaks around −138.64, −141.3, and −160.34 ppm were related to C-F bonds after random polymerization. The presence of asymmetric structures due to the flexible rotation of the chains was indicated by the observed duplication of peaks.

### 3.4. Thermal Stability of the Polymers

The thermal stability of organic polymers is a crucial factor that determines their potential applications in various fields, including electronic and environmental applications. Perfluorinated polymers (PFPs) have shown promising properties in terms of thermal stability under both air and inert conditions, making them an attractive option for such applications [36]. A widely recognized fact is that the presence of a significant number of heteroatoms in polymer structures increases their thermal stability [37]. The newly synthesized polymers contain a high amount of fluorine and nitrogen functional groups, which make them potentially thermally stable. Therefore, it was important to investigate the thermal stability of these polymers.

**Figure 4:**
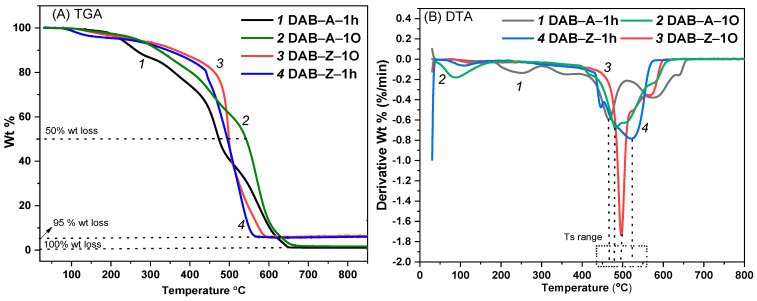
(**A**) Thermal gravimetric analysis (TGA) thermograms of the polymers; (**B**) first derivative (DTG) of the polymers.

The thermal stability of the newly synthesized polymers was investigated using TGA with a heating rate of 10 °C/min from 30 to 850 °C under an air atmosphere, as shown in Figure 4A. The TGA results indicate that DAB-Z-1h and DAB-Z-1O exhibit significantly higher thermal stability when compared to DAB-A-1h and DAB-A-1O. The higher thermal stability observed in the diazo-based polymers could be attributed to their high nitrogen content as well as the para-connections between the linkers and cross-linking. It has been suggested in several studies that para-connected polymers exhibit greater thermal stability than their meta- or ortho-connected counterparts [38]. In contrast, DAB-A-1O exhibited higher thermal stability than DAB-A-1h, possibly due to its high fluorine content and linear chain formation, as verified by the ^19^F NMR analysis. This finding is consistent with a previously reported series of linear and para-connected dibenzoxane-fluorinated chains, where the most stable chains were rigid-rod polymer chains [11].

To investigate the thermal stability of the polymers, DTG curves were generated (Figure 4B). The curves showed sevral distinct stages of decomposition varied in the range of 450–600 °C, which represents the peak temperature at which the materials decompose at the maximum rate (Ts). At 100 °C, there is no indication of the presence of water or accumulated solvents, except for an obvious trace in the case of DAB-A-1O, which might be removed with adequate drying time. The Ts value was found to be approximately 500 °C for DAB-Z-1h, DAB-Z-1O, and 484 °C for DAB-A-1O, while it was around 461 °C for DAB-A-1h. These results suggest that the high nitrogen content in the diazo-based polymers contributes to their superior thermal stability. Furthermore, the higher Ts value observed for DAB-A-1O compared to DAB-A-1h further supports the role of linear chains and fluorine in enhancing thermal stability [11].

Table 2 summarizes the temperatures of decomposition at 50% and 100% weight loss, as well as the Ts values, for all the polymers studied. At 100% weight loss, DAB-A-1h and DAB-A-1O were completely decomposed, leaving only residual ash due to the oxidative process. On the other hand, DAB-Z-1h and DAB-Z-1O were decomposed down to 95% of their contents, leaving a non-oxidized sample, which might be attributed to the high nitrogen load when compared with the 100% decomposed samples. The TGA/DTG data indicated that the diazo-based polymers exhibited higher thermal stability than the azomethine-based polymers, which could be attributed to their high nitrogen content. Additionally, the higher thermal stability of DAB-A-1O compared to DAB-A-1h may be due to the linear chain formation and high fluorine content.

### 3.5. Porosity Measurements

**Scheme 3:**
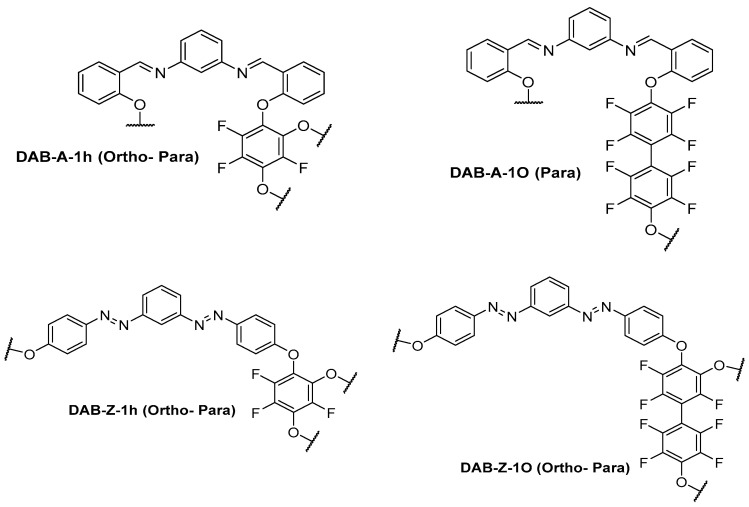
Possible structures of the polymers.

Based on the ^19^F NMR spectra, it was observed that DAB-A-1h, DAB-Z-1h, and DAB-Z-1O were formed as cross-linked chains, whereas DAB-A-1O was formed as an open chain, as summarized in Scheme 3. The formation of cross-linked chains is significant as it allows for the expansion of chains in two dimensions, resulting in an increased surface area and the formation of pores and cavities within the polymer chains.

**Figure 5:**
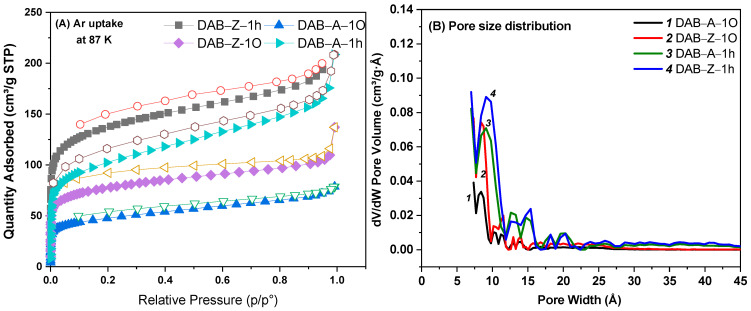
(**A**) Argon uptake isotherms at 87 K (closed symbols: adsorption, empty symbols: desorption); (**B**) pore size distribution.

To assess the potential formation of porous chains, the textural properties of the materials were analyzed using argon sorption measurements at 87 K (as shown in Figure 5A). The isotherms show the physisorption (adsorption-desorption) process, where the closed and open symbols represent the adsorption and desorption steps, respectively. The adsorption shows the affinity of the gas molecules to cover the surface and enter inside the pores of the porous material, while the desorption represents a reversible departure of the gas from the surface and pores of the solid adsorbent. Thus, the feasibility of gas removal from the surface and pores is practically known as a sample regeneration step. The argon adsorption-desorption isotherms showed large uptake at low relative pressure (0–0.1 bar), indicating permanent microporosity, and the isotherms were fully reversible [39,40,41]. 

The microporous nature of the porosity of the polymers was confirmed by the pore size distribution (PSD) curve obtained from the nonlocal density functional theory (NLDFT), which showed a peak centered in the range of 5–10 Å (Figure 5B) [42]. The pore volume of the polymers was measured at a relative pressure of P/P0 = 0.98 atm. The pore volume of each polymer was rationalized based on its surface area. Higher surface area values were associated with greater pore volumes. Since all polymers are conjugated chains, they are characterized by π-π stacking and tight packing, which decrease the measured porosity and limit the available free volume.

**Figure 6:**
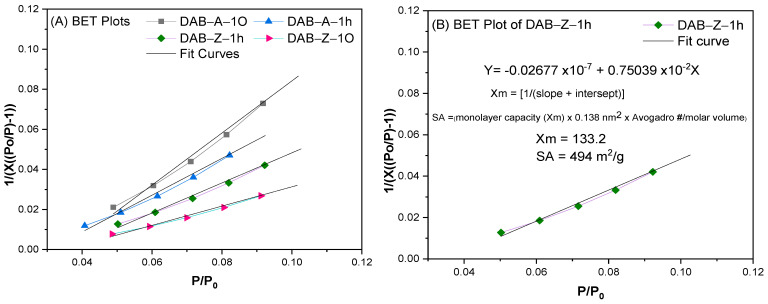
(**A**) BET multi-point fitting; and (**B**) determination of the BET of DAB-Z-1h. (X is the weight of gas adsorbed at relative pressure (P/P_0_)).

The surface area was determined using the Brunauer–Emmett–Teller (BET) method and by applying multi-point BET calculations. The calculations were performed on the gas adsorbed at a low pressure range of P/P_0_ between 0.025 and 0.30 [41]. The results of the BET determination are shown in Figure 6A, along with one sample calculation in Figure 6B, and summarized in Table S2. Further explanation of the calculations and the raw data are shown in Supplementary Part 2. 

As stated previously, the cross-linking in the polymers can create branching and increase their surface area. The open-chain polymer, DAB-A-1O, has the lowest surface area, measuring at 285 m^2^g^−1^, due to its lack of cross-linking. The low surface area of DAB-A-1O can be attributed to the steric effect, resulting in a partial structural collapse that limits the accessible sites for argon gas interactions. However, the surface areas of DAB-A-1h, DAB-Z-1h, and DAB-Z-1O were higher, determined as 403, 494, and 770 m^2^g^−1^, respectively. The observed surface area values for the synthesized polymers were considered reasonable due to the formation of random cross-linked chains. It was observed that the HFB-based polymers had higher surface area values than the DFB-based polymers, which could be attributed to the presence of flexible and smaller HFB linkers that facilitate extended branching. It is noteworthy that the fluorinated polymers had higher surface area values compared to their non-fluorinated counterparts, as reported in a previous study on a series of fluorinated and non-fluorinated polyaminal polymers, which also showed higher N_2_, CH_4_, and CO_2_ uptake [43].

### 3.6. Environmental Applications

The newly synthesized polymers exhibit a range of desirable properties, such as tailored hydrophobicity (e.g., solubility in polar or non-polar solvents) and porosity, as described in the previous sections. These properties make them promising candidates for various environmental applications. Therefore, this section will assess their potential for use in specific environmental applications, including their ability to separate carbon dioxide, methane, phenol, and benzene from their media. These pollutants are of concern as carbon dioxide and methane are air pollutants, while phenol and benzene are water pollutants. The experimental procedures and the results obtained for these pollutants will be discussed in the following sections.

#### 3.6.1. Methane and Carbon Dioxide Adsorption

The increasing release of carbon dioxide, commonly known as “greenhouse gas,” into the atmosphere is a significant global environmental concern [44]. Approximately 85% of CO_2_ emissions originate from the combustion of oil, coal, and natural gas, particularly in developing countries due to their economic and industrial growth [45]. Therefore, addressing this issue and mitigating the contribution of CO_2_ to global warming is of utmost importance. Methods used to control CO_2_ emissions include ethanol-amine technology and solid sorbents, such as MOFs (Metal Organic Frameworks). However, these methods have limitations, such as the high energy required for CO_2_ recovery from ethanolamine solutions and the air sensitivity of MOFs. Porous organic polymers (POPs) are a well-established category of materials designed for this purpose, owing to their air stability, high gas capture and storage capacity, and physisorption mechanism for CO_2_ desorption [46,47].

Methane (CH_4_) is a constituent of natural gas and is the second most significant greenhouse gas after CO_2_ [48]. Due to its abundance and lower carbon emissions, methane is considered an alternative to petroleum fuel. Therefore, there is significant interest in replacing gasoline and petroleum with methane, and many energy agencies and research groups are developing porous adsorbents capable of capturing it from the environment or gas mixtures. Zeolites, activated carbon, and metal organic frameworks (MOFs) are some examples of these adsorbents that require high pressure for methane capture but can be performed at room temperature, providing economic benefits, convenience, and high safety [49].

**Figure 7:**
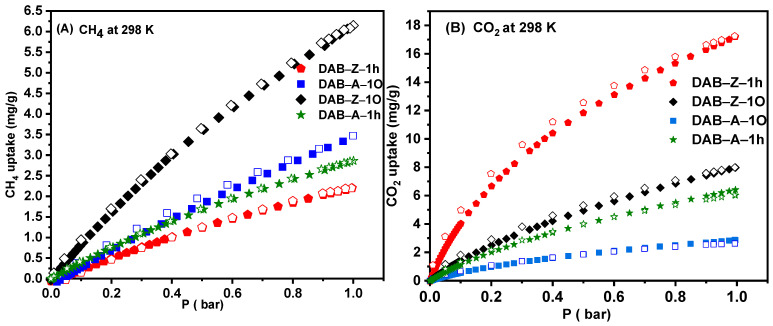
(**A**) CH_4_ gas uptake by the polymers at 298 K; (**B**) CO_2_ gas uptake by the polymers at 298 K.

The primary challenge in achieving efficient methane uptake is the need for a suitable surface area with an optimal pore size and functionalized surface that facilitates physical adsorption processes. Due to methane’s lack of a dipole moment, high pressure is required to pack the gas molecules within the pores of high-surface-area polymers. This high-pressure requirement presents safety challenges, making it crucial to design and develop materials that can safely and effectively capture methane [49]. In this section, we investigate the potential of the fluorinated polymers’ hydrophobicity and porosity for the separation of non-polar CH_4_ and polarizable CO_2_ gases (Figure 7) as an alternative to high-pressure methods. The gas uptake isotherms for CH_4_ and CO_2_ were measured at 298 K, which corresponds to real-world environmental conditions.

Based on the CH_4_ uptake values presented in Table 3 and Figure 7, DAB-Z-1O and DAB-A-1O exhibit uptake values of 6.14 and 3.46 mg/g, respectively. These values are higher than those of DAB-Z-1h and DAB-A-1h, which can be attributed to the incorporation of decafluorobiphenyl with a high fluorine content. Other studies have also confirmed that fluorinated materials have superior CH_4_ uptake, such as the fluorinated MOF NOTT-108a, which shows higher CH_4_ uptake compared to non-fluorinated MOF NOTT-101a [50]. 

**Table 3:** BET surface area, pore volume, and CH_4_ and CO_2_ uptake values of the polymers.

Polymer	Surface Area (BET) (m^2^ g^−1^)	Total Pore Volume (cm^3^/g)	CH_4_ Uptake (mg/g)	CO_2_ Uptake (mg/g)	Selectivity CO_2_/CH_4_
DAB-A-1h	403	0.2103	2.86	6.0	4.19
DAB-A-1O	285	0.0872	3.46	2.6	1.2
DAB-Z-1h	494	0.3524	2.19	17.2	14.9
DAB-Z-1O	770	0.2587	6.14	7.98	1.64

**Figure 8:**
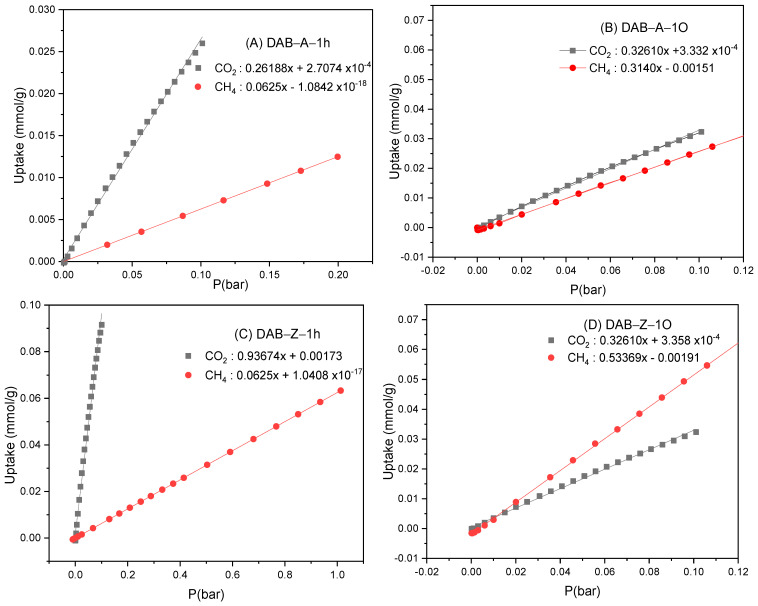
The initial slope calculations for DAB-A-1h (**A**), DAB-A-1O (**B**), DAB-Z-1h (**C**), and (**D**) DAB-Z-1O at 298 K.

BET: Brunauer–Emmett–Teller/Total pore volume calculated at p/p_0_ = 0.98; CH_4_ and CO_2_ uptake at 298 K. Selectivity (CO_2_/CH_4_) is determined from slope ratios (see Figure 8).

The CO_2_ uptake values of the polymers were also investigated, and the results showed that DAB-Z-1h exhibited the highest uptake (17.21 mg/g) due to its fairly high porosity and nitrogen content compared to the other polymers. Therefore, controlling the hydrophobicity and porosity of the polymers could be crucial factors that determine their gas uptake capabilities [51]. Fluorine-rich polymers with low surface areas are preferred for CH_4_ uptake, while nitrogen-rich polymers with high surface areas are preferred for CO_2_ uptake.

To assess the ability of the new polymers as adsorbents for CO_2_ or CH_4_, we calculated a selectivity parameter. Various calculation methods can be used to determine the selective binding of CO_2_/CH_4_ at 298 K, such as (1) initial slope calculations, (2) Henry’s Law constants, and (3) ideal adsorbed solution theory (IAST) [52]. Herein, we utilized the initial slope method to determine the selectivity parameter for CO_2_ or CH_4_ adsorption. This method involves the calculation of the slope of the linear relationship between the low-pressure range and gas uptake (mmol g^−1^) in the pressure range of 0–0.1 bar. The slope ratio between the pure gas isotherms of CO_2_ and CH_4_ at 298 K provides the numerical selectivity value. Figure 8 illustrates the calculation process by taking the slope of each CO_2_ and CH_4_ pure gas isotherm at 298 K in the low-pressure range and then computing the slope ratio between the pure gas isotherms [53]. The selectivity results for CO_2_/CH_4_ are presented in Table 3, which shows that the polymers with the highest CO_2_ uptake are also the most selective for CO_2_. Conversely, DAB-A-1O and DAB-Z-1O, which are rich in fluorine, exhibit a higher binding selectivity for CH_4_ than CO_2_.

#### 3.6.2. Separation of Benzene–Water and Phenol–Water Mixtures

The fluorine and nitrogen contents, as well as the tailored polarity of the polymers, are factors that facilitate the separation of polar/polarizable CO_2_ and non-polar CH_4_ molecules from the air. The potential of these polymers for separating other organic molecules from water was also examined. The selected solvents were benzene (non-polar) and phenol (polar), and the separation process was carried out. In line with the aforementioned hypothesis, the polymers with a high fluorine loading exhibited a significant hydrophobic nature, which makes them promising adsorbents for benzene. A similar study has used fluorinated polyethers supported by graphene oxide surfaces for the separation of oil and water [54]. Conversely, the cross-linked polymers with low fluorine contents contain reactive C-F bonds and high nitrogen contents, making them potentially effective adsorbents for phenol as a polar solvent. To perform qualitative adsorption experiments, the polymers were submerged in benzene-water or phenol-water-saturated solutions for a certain period. UV–Vis absorption measurements of the solutions were then conducted over a 90 min period to track the amount of organic solvent adsorbed onto the polymer surfaces.

**Figure 9:**
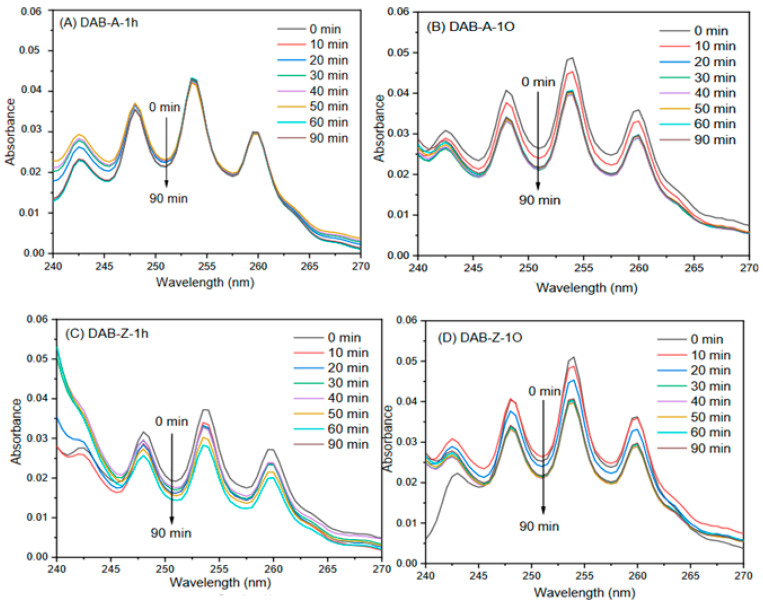
The UV–visible spectra of benzene adsorption by polymers over a period of time.

Figure 9A shows the UV–Vis absorption of the benzene-water solution. DAB-A-1h displayed a consistent trend for benzene adsorption throughout the measurement time. This behavior can be attributed to its hydrophilic nature due to free C-F and nitrogen. Additionally, the ortho-connected structure of DAB-A-1h facilitated the formation of an agglomerated and collapsed structure, which led to a reduction in surface area and a subsequent limitation in the ability of benzene to be adsorbed on the surface or within the pores. DAB-A-1O exhibited a more distinct trend for benzene separation from water in the initial measurement after 10 min, as shown in Figure 9B. This behavior suggests that the adsorption of benzene on the octafluorobiphenyl linker occurred as a one-layer adsorption with time. The para-open extended chain and the ortho-para-branched framework of the diazo-based polymers (DAB-Z-1h and DAB-Z-1O) were confirmed to form cross-linked structures through the ^19^F NMR spectra. Moreover, the azo-groups in these polymers were found to be para-related to the ether C-O-C bond (as depicted in Scheme 3), which facilitated the rate of nucleophilic aromatic substitution (NAS) and promoted the flexible rotation of the chains. This, in turn, increased the rate of interaction with the solvent, as demonstrated in Figure 9C,D. The initial response of these polymers towards benzene adsorption during the first 30 min can be attributed to the presence of accessible C-F sites that possess a diminished dipole moment Figure S15.

**Figure 10:**
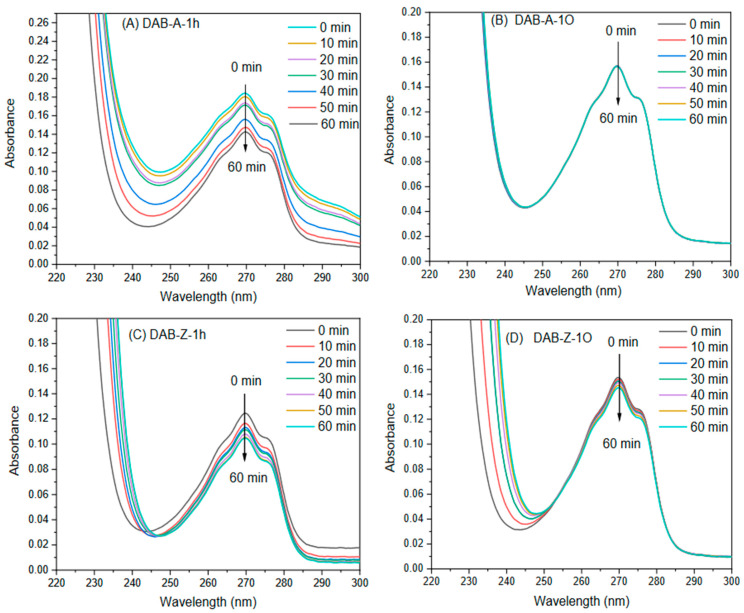
The UV–visible spectra of phenol adsorption by polymers over a period of time.

Phenol is a harmful polar organic pollutant commonly found in water, posing a significant threat to both humans and aquatic life [54]. Thus, its presence in the water must be restricted. Sources of phenol pollution in water include industrial waste from oil refineries, coal tar, and plastics, as well as waste from olive farming and agricultural activities, in addition to its natural occurrence [55,56]. The impact of connection sites, cross-linking, and flexibility on the polarities of the polymers DAB-A-1h, DAB-A-1O, DAB-Z-1h, and DAB-Z-1O enabled us to explore their potential in adsorbing benzene. To provide a comparative study, we also evaluated the ability of these polymers to separate phenol from water through similar experimental procedures used in the benzene adsorption experiments. As previously demonstrated, DAB-A-1O exhibited a highly hydrophobic nature and showed potential for adsorbing benzene, as seen in the previous experiment. This observation was further confirmed by the decrease in adsorption of phenol on the polymer surface, as shown in Figure 10A. Additionally, the presence of octafluorobiphenyl in DAB-Z-1O supports the observation of a low response to phenol adsorption, as shown in Figure 10B. In contrast, DAB-A-1h and DAB-Z-1h, which were formed through the reactive NAS and have low fluorine and high nitrogen contents, a moderate surface area, and flexible chains, showed improved interactions with phenol, as depicted in Figure 10C,D.

## 4. Conclusions

In this study, four new fluorinated organic polymers using the nucleophilic aromatic substitution (NAS) reaction were synthesized and fully characterized. The polymers are thermally stable, and their possibility of forming a porous analog was confirmed by ^19^F NMR. The surface area of the polymers, as measured by argon sorption, ranged from 285 to 770 m^2^/g. Due to their characteristics, including the presence of fluorine, nitrogen, pores, and cavities, along with a reasonable surface area, this new series of polymers is a promising candidate for separation applications. Polymers with a high concentration of fluorine are more hydrophobic, making them suitable for separating methane gas and benzene from media. The combination of hydrophobicity and controlled cross-linking in the design of fluorinated polymers makes this class of polymers vital for environmental remediation. High-surface-area polymers with high nitrogen contents are suitable for CO_2_ capture and phenol separation from media, while polymers with high fluorine concentrations are applicable for separating methane gas and benzene from media. The polyers have shown tailored selectivity toward CO_2_/CH_4_ adsorption and benzene/phenol separation. Following the synthesis of these polymers, our future work will focus on studying the mechanisms behind these separation processes by determining their adsorption kinetics.

## Figures and Tables

**Table 1:** Physical properties of the polymers.

Polymer	Appearance	Solubility	% Yield	Mn (g/mol)	Mwt (g/mol)
DAB-Z-1h	Dark brown	DMSO ^a^, DMAc ^a^, THF ^c^	88	insoluble in THF	
DAB-Z-1O	Dark brown	DMSO, DMAc, THF ^a^	70	3106	7328
DAB-A-1h	Pale brown	DMSO, DMAc, THF ^a^	82	1381	4886
DAB-A-1O	Pale brown	THF ^b^, DMSO ^c^, DMAc ^c^	69	4068	11,948

(^a^) slightly soluble; (^b^) completely soluble; (^c^) insoluble; Mn: number, average molecular weight; Mwt: weight, average molecular weight.

**Table 2:** Data extrapolated from TGA and DTA.

Polymer	Decomposition (°C)	Ts (DTA)	T (°C) 50%	T (°C) 100%	T (°C) 95%
DAB-Z-1h	420	498	500	-	590
DAB-Z-1O	423	521	493	-	566
DAB-A-1h	230	461	471	649	-
DAB-A-1O	283	484	544	661	-

DTA: first derivative of TGA; Ts: the peak temperature derived from the DTA curve; (-): no data.

## Data Availability

The data presented in this study are available on request from the corresponding author.

## Supplementary Materials

The following supporting information can be downloaded at: https://www.mdpi.com/article/10.3390/polym15204191/s1, Scheme S1: Proposed mechanism of the nucleophilic aromatic substitution reaction (NAS); Table S1: Reported values of the chemical shift of ^19^F NMR and different sites; Figures S1–S4: 1H and ^13^C NMR Spectra of the monomers; Figures S5–S12: ^1^H and ^13^C NMR Spectra of the polymers; Figure S13: Solubility behavior of the polymers in THF and DMSO; Figure S14: SEM images of the polymers; Figure S15: Proposed sites of zero and non-zero dipole moments of the polymers; Part 2: BET surface area determination; Table S2: BET plots data of the polymers.

## References

[1] Zhou J., Tao Y., Chen X., Chen X., Fang L., Wang Y., Sun J., Fang Q. (2019). Perfluorocyclobutyl-based polymers for functional materials. Mater. Chem. Front..

[2] Francesco B., Gianluca M.F., Francesco N., Roberta R. (2007). Fluorinated organic materials for electronic and optoelectronic applications: The role of the fluorine atom. Chem. Commun..

[3] Kenji M., Kenichi O., Eishun T., Allan S.H. (2001). Synthesis and properties of novel sulfonated arylene ether/fluorinated alkane copolymers. Macromolecules.

[4] Tkachenko I.M., Kurioz Y.I., Kovalchuk A.I., Kobzar Y.L., Shekera O.V., Tereshchenko O.G., Nazarenko V.G., Shevchenko V.V. (2020). Optical properties of azo-based poly(azomethine)s with aromatic fluorinated fragments, ether linkages and aliphatic units in the backbone. Mol. Cryst. Liq..

[5] Ling Y., Rongming X., Hang Y., Chenghan J., Lu L., Weiming Z. (2023). Three-dimensional fluorinated pyrazinium-based cationic organic polymers with high charge density for enhanced ReO_4_^−^ removal. J. Chem. Eng..

[6] Gouverneur V., Seppelt K. (2015). Introduction: Fluorine Chemistry. Chem. Rev..

[7] Errifai I., Jama C., Le Bras M., Delobel R., Mazzah A., Roger J. (2004). Fire retardant coating using cold plasma polymerization of a fluorinated acrylate. Surf. Coat. Technol..

[8] Rowe M., Teo G.H., Horne J., Al-Khayat O., Neto C., Thickett S.C. (2016). High Glass Transition Temperature Fluoropolymers for Hydrophobic Surface Coatings via RAFT Copolymerization. Aust. J. Chem..

[9] Tundidor-Camba A., González-Henríquez C.M., Sarabia-Vallejos M.A., Tagle L.H., Hauyón R.A., Sobarzo P.A., González A., Ortiz P.A., Maya E.M., Terraza C.A. (2018). Silylated oligomeric poly(ether-azomethine)s from monomers containing biphenyl moieties: Synthesis and characterization. RSC Adv..

[10] Zhou J., Liu J., Wang M., Hou W., Qin G., Kityk I.V., Fedorchuk A.A., Albassam A.A., El-Naggar A.M., Andrushchak A. (2017). Novel poly(aryl ether ketone) with electro-optic chromophore side chains for light modulators. J. Mater. Sci. Mater. Electron..

[11] Sijing C., Dengxun R., Bo L., Kui L., Lin C., Mingzhen X., Xiaobo L. (2019). Benzoxazine Containing Fluorinated Aromatic Ether Nitrile Linkage: Preparation, Curing Kinetics and Dielectric Properties. Polymers.

[12] Yao W., He L., Han D., Zhong A. (2019). Sodium Triethylborohydride-Catalyzed Controlled Reduction of Unactivated Amides to Secondary or Tertiary Amines. J. Org. Chem..

[13] Liu Q., Shentu B., Zhu J., Weng Z. (2007). A novel synthetic method for preparing poly(2,6-dimethyl-1,4-phenylene oxide)/polystyrene alloy in reactor containing aqueous medium. Eur. Polym. J..

[14] Jennifer A.I., Charles J.N., Kevin M.K., Patrick E.C., Anne K.S. (1992). Polyethers derived from bisphenols and highly fluorinated aromatics. J. Polym. Sci. Part A Polym. Chem..

[15] Krishnan R., Parthiban A. (2014). Regioselective preparation of functional aryl ethers and esters by stepwise nucleophilic aromatic substitution reaction. J. Fluor. Chem..

[16] Wall L.A., Pummer W.J., Fearn J.E., Antonucci J.M. (1963). Reactions of Polyfluorobenzenes with Nucleophilic Reagents. J. Res. Natl. Bur. Stand A Phys. Chem..

[17] Wang Z., Shang Y., Han X., Yan Q., Liu J., Jiang Z., Zhang H. (2020). Cross-Linked Fluorinated Poly(Aryl Ether) (C-FPAE) Films: Preparation Strategy, Performance Study, and Low Dielectric Applications. Macromol. Mater. Eng..

[18] Ando S., Matsuura T., Sasaki S. (2006). Synthesis and properties of perfluorinated polyimides. Topics in Applied Chemistry.

[19] Chen Y.C., Su Y.Y., Hsiao F.Z. (2020). The synthesis and characterization of fluorinated polyimides derived from 2′-methyl-1,4-bis-(4-amino-2-trifluoromethylphenoxy)benzene and various aromatic dianhydrides. J. Macromol. Sci. A.

[20] Li X., Gao Y., Long Q., Hay A.S. (2014). Synthesis and characterization of highly fluorinated poly(phthalazinone ether)s based on AB-type monomers. J. Polym. Sci. Part A Polym. Chem..

[21] Shundrina I.K., Vaganova T.A., Kusov S.Z., Rodionov V.I., Karpova E.V., Malykhin E.V. (2011). Synthesis and properties of organosoluble polyimides based on novel perfluorinated monomer hexafluoro-2,4-toluenediamine. J. Fluor. Chem..

[22] Ihor T., Olha P., Valery B., Oleg S., Valery S. (2015). Synthesis and properties of novel fluorinated poly(arylene ether)s. Polym. Int..

[23] Zhao T., Beyer V.P., Becer C.R. (2020). Fluorinated Polymers via Para-Fluoro-Thiol and Thiol-Bromo Click Step Growth Polymerization. Macromol. Rapid Commun..

[24] Yao W., Wang J., Zhong A., Li J., Yang J. (2020). Combined KOH/BEt3 Catalyst for Selective Deaminative Hydroboration of Aromatic Carboxamides for Construction of Luminophores. Org. Lett..

[25] Narayanan G., Faradizaji B., Mukeba K.M., Shelar K.E., De Silva M., Patrick A., Donnadieu B., Smith D.W. (2020). Perfluorocyclohexenyl (PFCH) aromatic ether polymers from perfluorocyclohexene and polycyclic aromatic bisphenols. Poly. Chem..

[26] Smirnova O., Glazkov A., Yarosh A., Sakharov A. (2016). Fluorinated Polyurethanes, Synthesis and Properties. Molecules.

[27] Ong W.J., Swager T.M. (2018). Dynamic self-correcting nucleophilic aromatic substitution. Nat. Chem..

[28] Zhang T., Yao N., Zhou C., Li Y., Zhao Y., Pang A., Wu S. (2022). Combustion characteristics of cross-linked fluorinated polymer supported aluminum/oxidizer microsphere in HTPB propellant. FirePhysChem.

[29] Mohammadi A., Jabbari J. (2016). Simple naked-eye colorimetric chemosensors based on Schiff-base for selective sensing of cyanide and fluoride ions. Can. J. Chem..

[30] Mohammadi A., Yazdanbakhsh M.R., Farahnak L. (2012). Synthesis and evaluation of changes induced by solvent and substituent in electronic absorption spectra of some azo disperse dyes. Spectrochim. Acta-A Mol. Biomol. Spectrosc..

[31] Aivali S., Andrikopoulos K.C., Andreopoulou A.K. (2023). Nucleophilic Aromatic Substitution of Pentafluorophenyl-Substituted Quinoline with a Functional Perylene: A Route to the Modification of Semiconducting Polymers. Polymers.

[32] Krishnan R., Parthiban A. (2013). Semifluorinated multiple pendant group bearing poly(arylene ether) copolymers prepared at room temperature. J. Polym. Res..

[33] Krishnan R., Parthiban A. (2018). A multifunctionalization of octafluorocyclopentene under mild conditions. Eur. Polym. J..

[34] Jaye J.A., Sletten E.M. (2021). Recent advances in the preparation of semifluorinated polymers. Polym. Chem..

[35] Kaseman D.C., Janicke M.T., Frankle R.K., Nelson T., Angles-Tamayo G., Batrice R.J., Magnelind P.E., Espy M.A., Williams R.F. (2020). Chemical Analysis of Fluorobenzenes via Multinuclear Detection in the Strong Heteronuclear J-Coupling Regime. Appl. Sci.

[36] Xiao F., Sasi P.C., Yao B., Kubátová A., Golovko S.A., Golovko M.Y., Soli D. (2020). Thermal Stability and Decomposition of Perfluoroalkyl Substances on Spent Granular Activated Carbon. Environ. Sci. Technol. Lett..

[37] Al-tarawneh S.S., Ababneh T., Aljaafreh I. (2021). Amination of ether-linked polymers via the application of Ullmann-coupling reaction: Synthesis, characterization, porosity, and thermal stability evaluation. Int. J. Polym. Anal..

[38] Shockravi A., Javadi A., Abouzari-Lotf E. (2011). Fluorinated ortho-linked polyamides derived from non-coplanar 1,1′-thiobis(2-naphthol): Synthesis and characterization. Polym. J..

[39] Bae Y.-S., Yazaydın A.Ö., Snurr R.Q. (2010). Evaluation of the BET Method for Determining Surface Areas of MOFs and Zeolites that Contain Ultra-Micropores. Langmuir.

[40] Merukan Chola N., Gajera P., Kulkarni H., Kumar G., Parmar R., Nagarale R.K., Sethia G. (2023). Sorption of Carbon Dioxide and Nitrogen on Porous Hyper-Cross-Linked Aromatic Polymers: Effect of Textural Properties, Composition, and Electrostatic Interactions. ACS Omega.

[41] Shi K., Li Z., Anstine D.M., Tang D., Colina C.M., Sholl D.S., Siepmann J.I., Snurr R.Q. (2023). Two-Dimensional Energy Histograms as Features for Machine Learning to Predict Adsorption in Diverse Nanoporous Materials. J. Chem. Theory Comput..

[42] Kupgan G., Liyana-Arachchi T.P., Colina C.M. (2017). NLDFT Pore Size Distribution in Amorphous Microporous Materials. Langmuir.

[43] Li G., Zhang B., Wang Z. (2016). Facile Synthesis of Fluorinated Microporous Polyaminals for Adsorption of Carbon Dioxide and Selectivities over Nitrogen and Methane. Macromolecules.

[44] Kabir M., Habiba U.E., Khan W., Shah A., Rahim S., De los Rios-Escalante P.R., Farooqi Z.U.R., Ali L., Shafiq M. (2023). Climate change due to increasing concentration of carbon dioxide and its impacts on environment in 21st century. King Saud Univ. Sci..

[45] Li J.R., Ma Y., McCarthy M.C., Sculley J., Yu J., Jeong H.K., Balbuena P.B., Zhou H.C. (2011). Carbon dioxide capture-related gas adsorption and separation in metal-organic frameworks. Coord. Chem. Rev..

[46] Wang H., Wang G., Hu L., Ge B., Yu X., Deng J. (2023). Porous Polymer Materials for CO_2_ Capture and Electrocatalytic Reduction. Materials.

[47] Al-Shboul T., Al-Tarawneh S., Ababneh T., Jazzazi T. (2022). Post-Functionalization of Bromo-Substituted Ether-Linked Polymers via Ullman Coupling Reaction: Synthesis, Characterization and Their Role toward Carbon Dioxide Capture. Separations.

[48] Li D., Chen L., Liu G., Yuan Z., Li B., Zhang X., Wei J. (2021). Porous metal-organic frameworks for methane storage and capture: Status and challenges. New Carbon Mater..

[49] Peng A.Y., Krungleviciute V., Eryazici I., Hupp J.T., Farha O.K., Yildirim T. (2013). Methane Storage in Metal–Organic Frameworks: Current Records, Surprise Findings, and Challenges. J. Am. Chem. Soc..

[50] Chang G., Wen H., Li B., Zhou W., Wang H., Alfooty K., Bao Z., Chen B. (2016). A Fluorinated Metal–Organic Framework for High Methane Storage at Room Temperature. Cryst. Growth Des..

[51] Mohammed S., Sunkara A.K., Walike C.E., Gadikota G. (2021). The Role of Surface Hydrophobicity on the Structure and Dynamics of CO_2_ and CH_4_ Confined in Silica Nanopores. Front. Clim..

[52] An J., Geib S.J., Rosi N.L. (2010). High and Selective CO_2_ Uptake in a Cobalt Adeninate Metal—Organic Framework Exhibiting Pyrimidine- and Amino-Decorated Pores. J. Am. Chem. Soc..

[53] Sekizkardes A.K., Altarawneh S., Kahveci Z., İslamoğlu T., El-Kaderi H.M. (2014). Highly Selective CO_2_ Capture by Triazine-Based Benzimidazole-Linked Polymers. Macromolecules.

[54] Liu C., Wei L., Jia X., Gu Y., Guo H., Geng X. (2023). Fluorinated-Polyether-Grafted Graphene-Oxide Magnetic Composite Material for Oil–Water Separation. AppliedChem.

[55] Hameed B.H., Rahman A.A. (2008). Removal of phenol from aqueous solutions by adsorption onto activated carbon prepared from biomass material. J. Hazard. Mater..

[56] Li H., Xu M., Shi Z., He B. (2004). Isotherm analysis of phenol adsorption on polymeric adsorbents from nonaqueous solution. J. Colloid Interface Sci..

