# Supplementary material for: Synthesis, Characterization, and Environmental Applications of Novel Per-Fluorinated Organic Polymers with Azo- and Azomethine-Based Linkers via Nucleophilic Aromatic Substitution
Source: Polymers (Basel). 2023 Oct 23;15(20):4191. doi: 10.3390/polym15204191 (PMC10610692; doi:10.3390/polym15204191)
Supplement: Supplementary file 1 [file polymers-15-04191-s001.zip › polymers-2651385-supplementary.pdf]

Synthesis, Characterization, and Environmental Applications of Novel Per-Fluorinated Organic Polymers with Azo- and Azomethine-Based Linkers via Nucleophilic Aromatic Substitution

Suha S. Altarawneh [1,*], Hani M. El-Kaderi [2], Alexander J. Richard [2], Osama M. Alakayleh [1], Ibtesam Y. Aljaafreh [1], Mansour H. Almatarneh [3], Taher S. Ababneh [4], Lo'ay A. Al-Momani [5] and Rawan H. Aldalabeeh [1]

[1] Department of Chemistry and Chemical Technology, Tafila Technical University, Tafila 66110, Jordan; osama.m.alakayleh@gmail.com (O.M.A.); ibtesam_aljaafreh@ttu.edu.jo (I.Y.A.); rawan.hanidalabeh@gmail.com (R.H.A.)
[2] Department of Chemistry, Virginia Commonwealth University, Richmond, VA 23284, USA; helkaderi@vcu.edu (H.M.E.-K.); richardaj@vcu.edu (A.J.R.)
[3] Department of Chemistry, University of Jordan, Amman 11942, Jordan; m.almatarneh@ju.edu.jo
[4] Department of Chemistry, Yarmouk University, Irbid 21163, Jordan; ababnehtaher@hotmail.com
[5] Department of Chemistry, Faculty of Science, The Hashemite University, Zarqa 13133, Jordan; loay.al-momni@hu.edu.jo
* Correspondence: s.tarawneh@ttu.edu.jo

Title	Page #
**Part S1:**<br>Scheme S1: Proposed mechanism of the nucleophilic aromatic substitution reaction (NAS).	**2**
Table S1: Reported values of the chemical shift of ^{19}F NMR and different sites.	**2**
Figures S1–S4: ^{1}H and ^{13}C NMR Spectra of the monomers.	**3–6**
Figures S5–S12: ^{1}H and ^{13}C NMR Spectra of the polymers.	**7–13**
Figure S13: Solubility behavior of the polymers in THF and DMSO.<br>Figure S14: SEM images of the polymers.	**14**
Figure S15: Proposed sites of zero and non-zero dipole moments of the polymers.	**15**
**Part S2:**<br>BET surface area determination.<br>Table S3: BET plots data of the polymers.	**15–16**
References	**16**

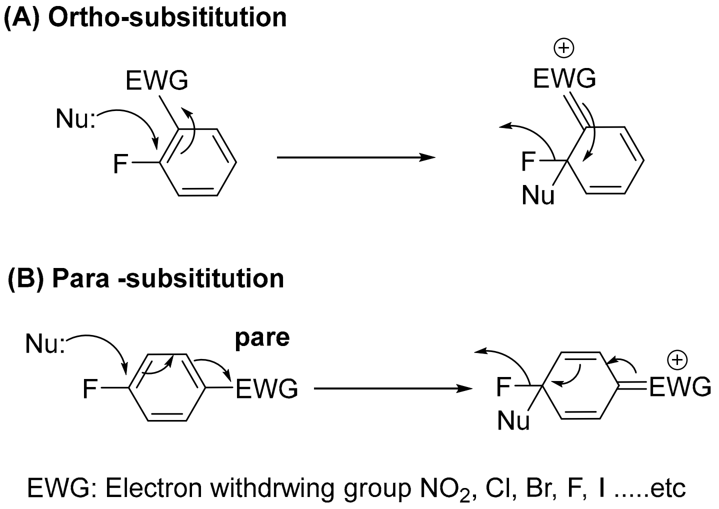

Scheme S1. Proposed mechanism of the nucleophilic aromatic substitution reaction (NAS).

Table S1. Reported values of the chemical shift of ^{19}F-NMR and different sites [1].

**Compound**	**Chemical Shift (ppm)**
**Benzene + Hexafluorobenzene**	-163.2
**Monofluorobenezne**	-112.9
**1,2-Difluorobenezne**	-138.4
**1,3-Difluorobenezne**	-109.9
**1,4-Difluorobenezne**	-119.6
	-143.5
**1,2,4-Trifluorobenzne**	-133.5
	-115.7
**1,2,4,5-Tetrafluorobenezne**	-139.7
	-162.5
**Pentafluorobenzene**	-154.2
	-139.2

List of ^{1}H- and ^{13}C-NMR Spectra of the monomers

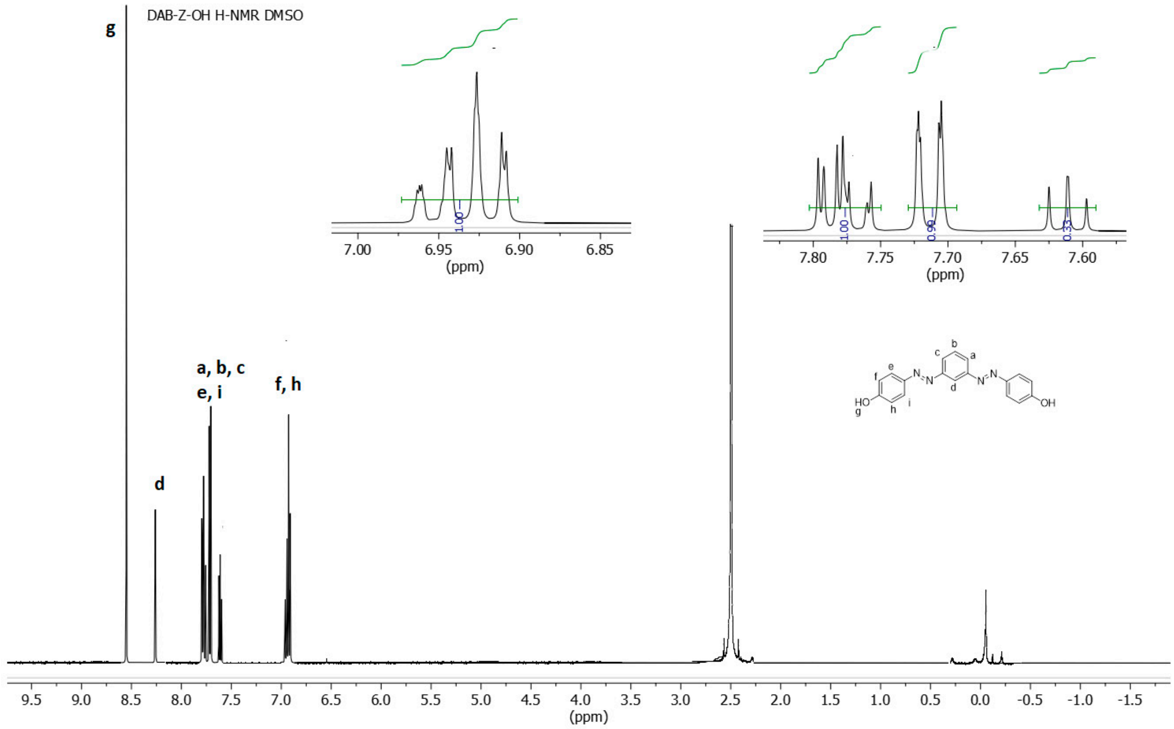

Figure S1. ^{1}H NMR spectra of DAB-Z-OH.

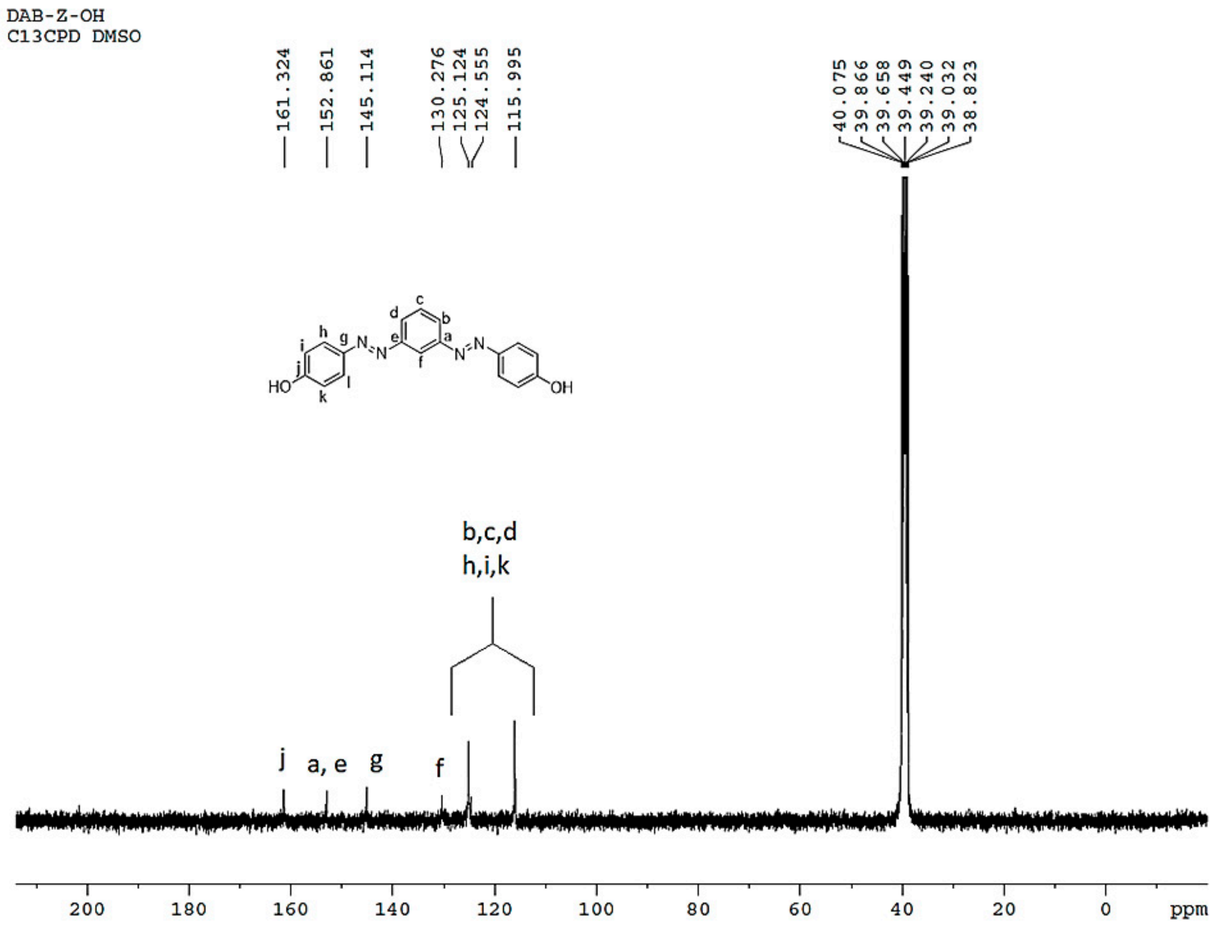

Figure S2. ^{13}C NMR spectra of DAB-Z-OH.

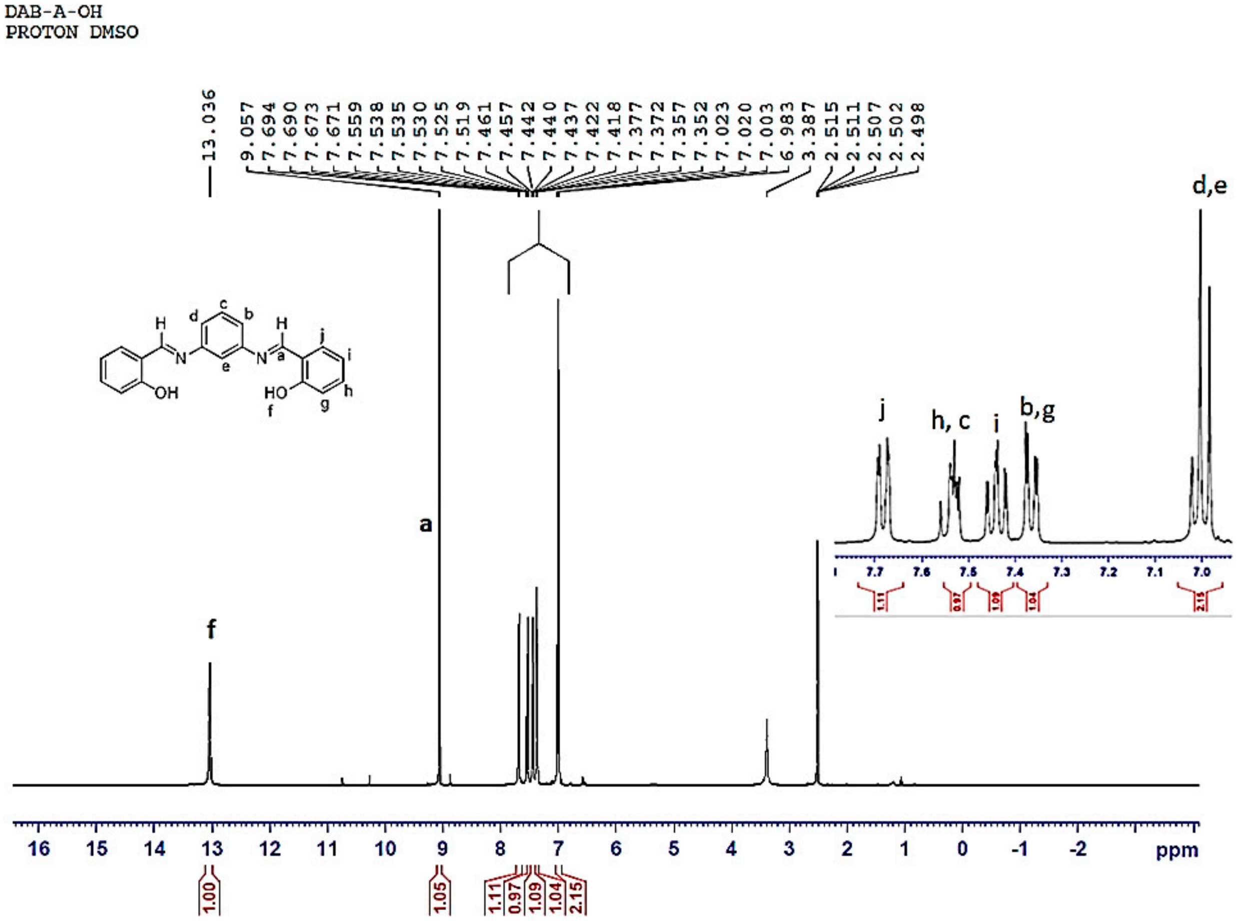

Figure S3. ^{1}H NMR spectra of DAB-A-OH.

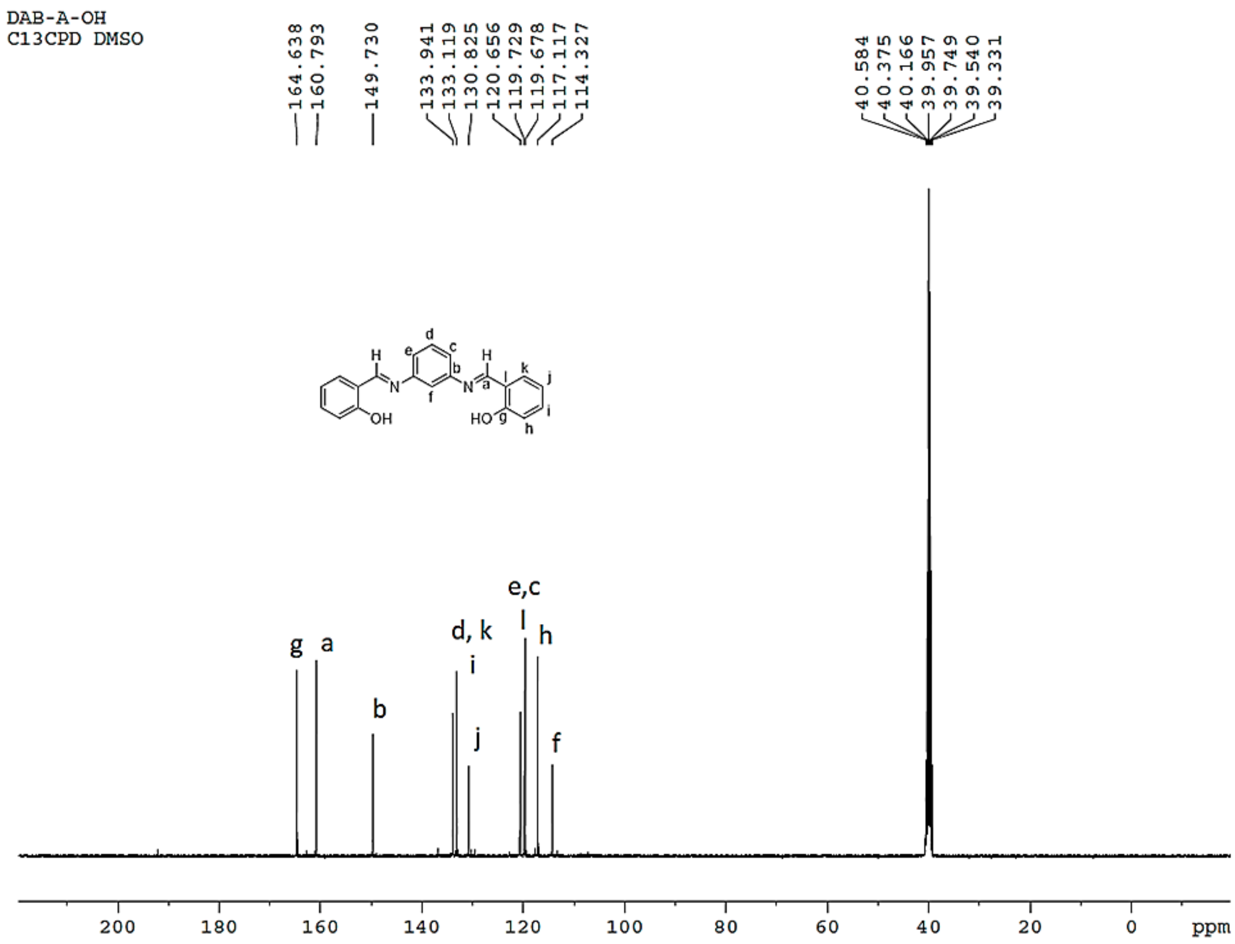

Figure S4. ^{13}C NMR spectra of DAB-A-OH.

^{1}H and ^{13}C NMR Spectra of the Polymers

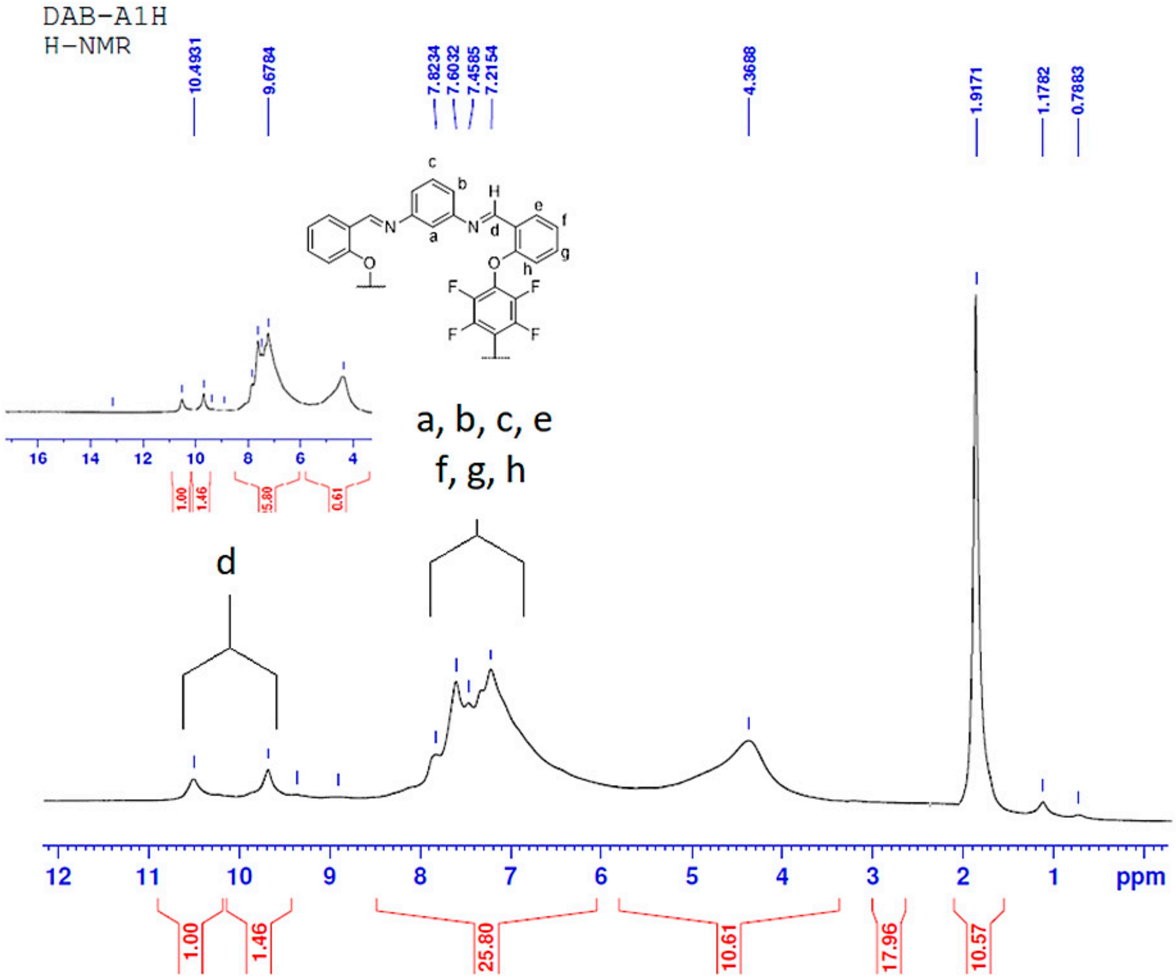

Figure S5. ^{1}H NMR spectra of DAB-A-1h.

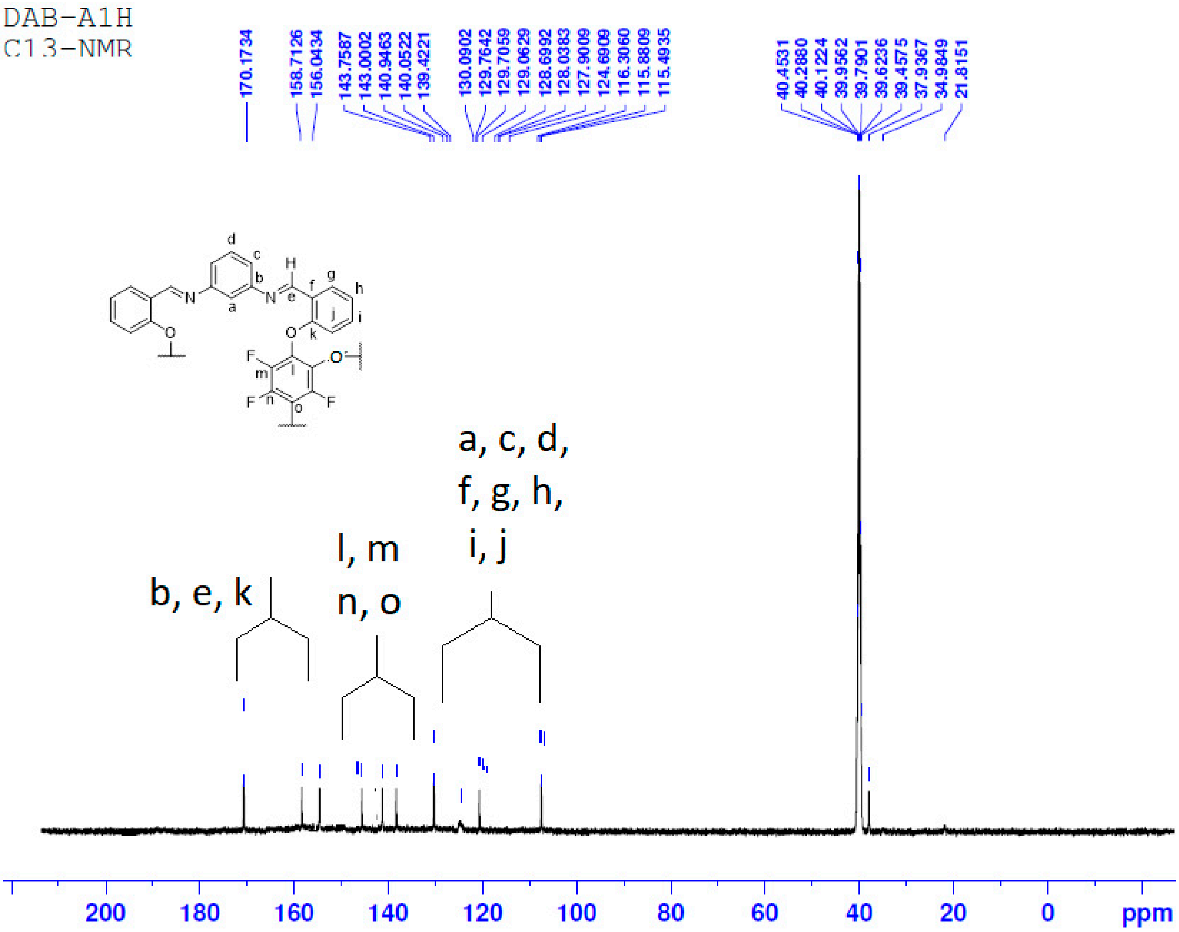

Figure S6. ^{13}C NMR spectra of DAB-A-1h.

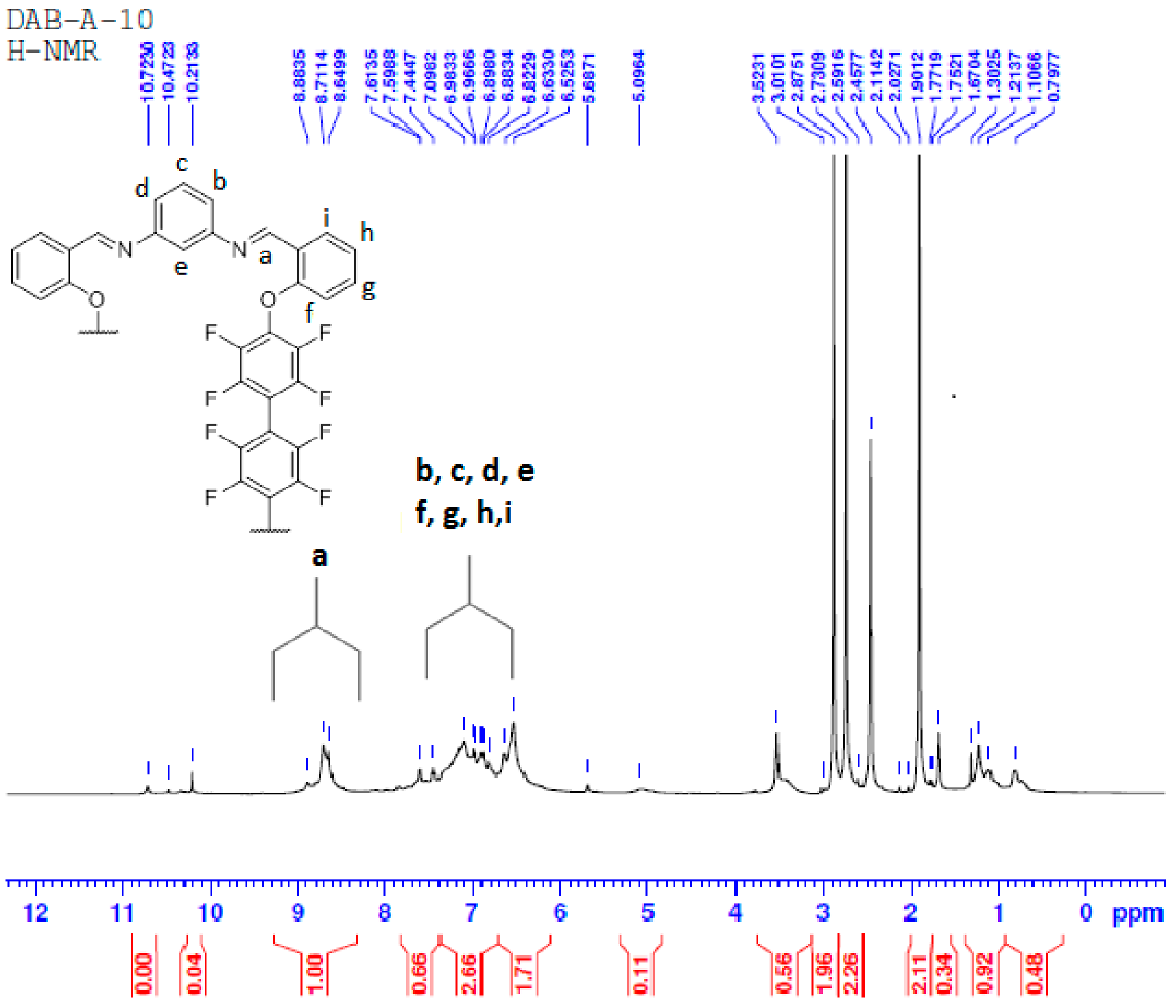

Figure S7. ^{1}H NMR spectra of DAB-A-1O.

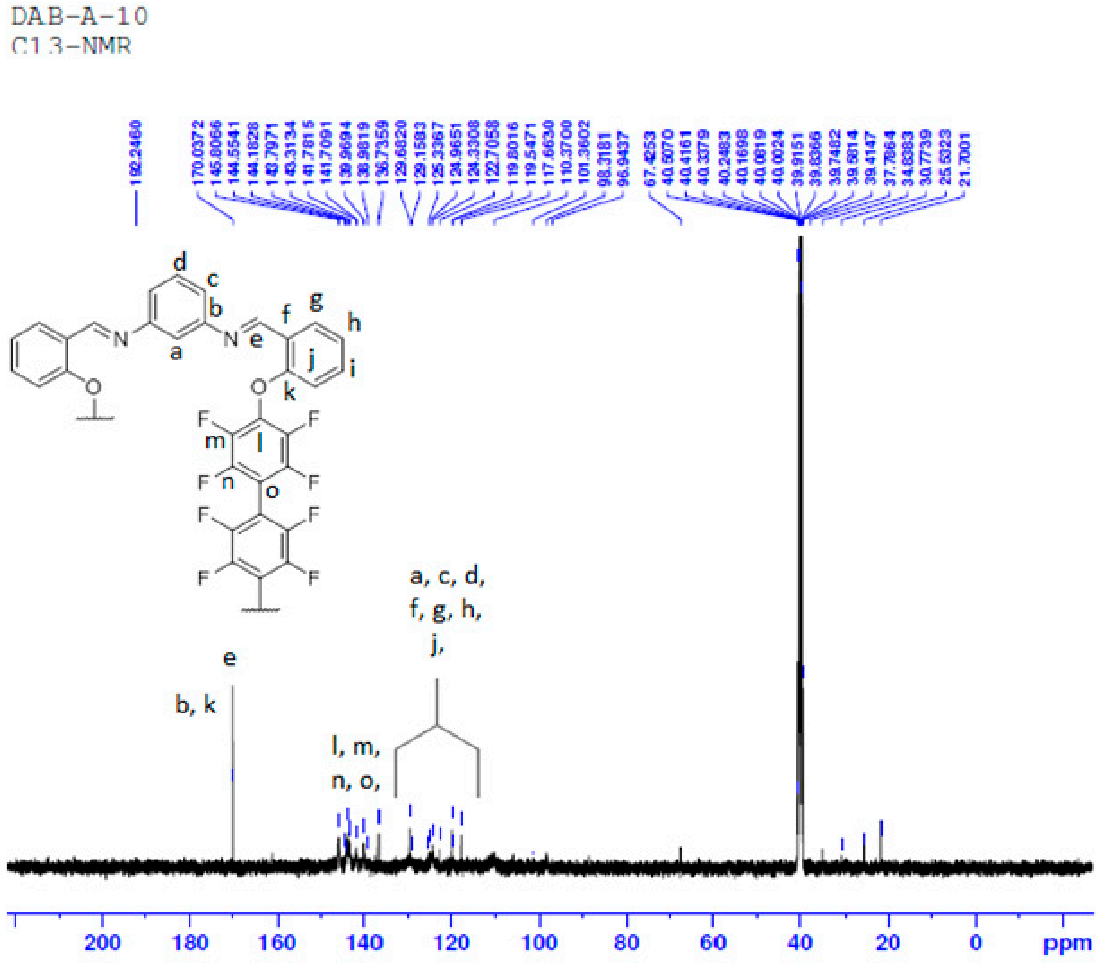

Figure S8. ^{13}C NMR spectra of DAB-A-1O.

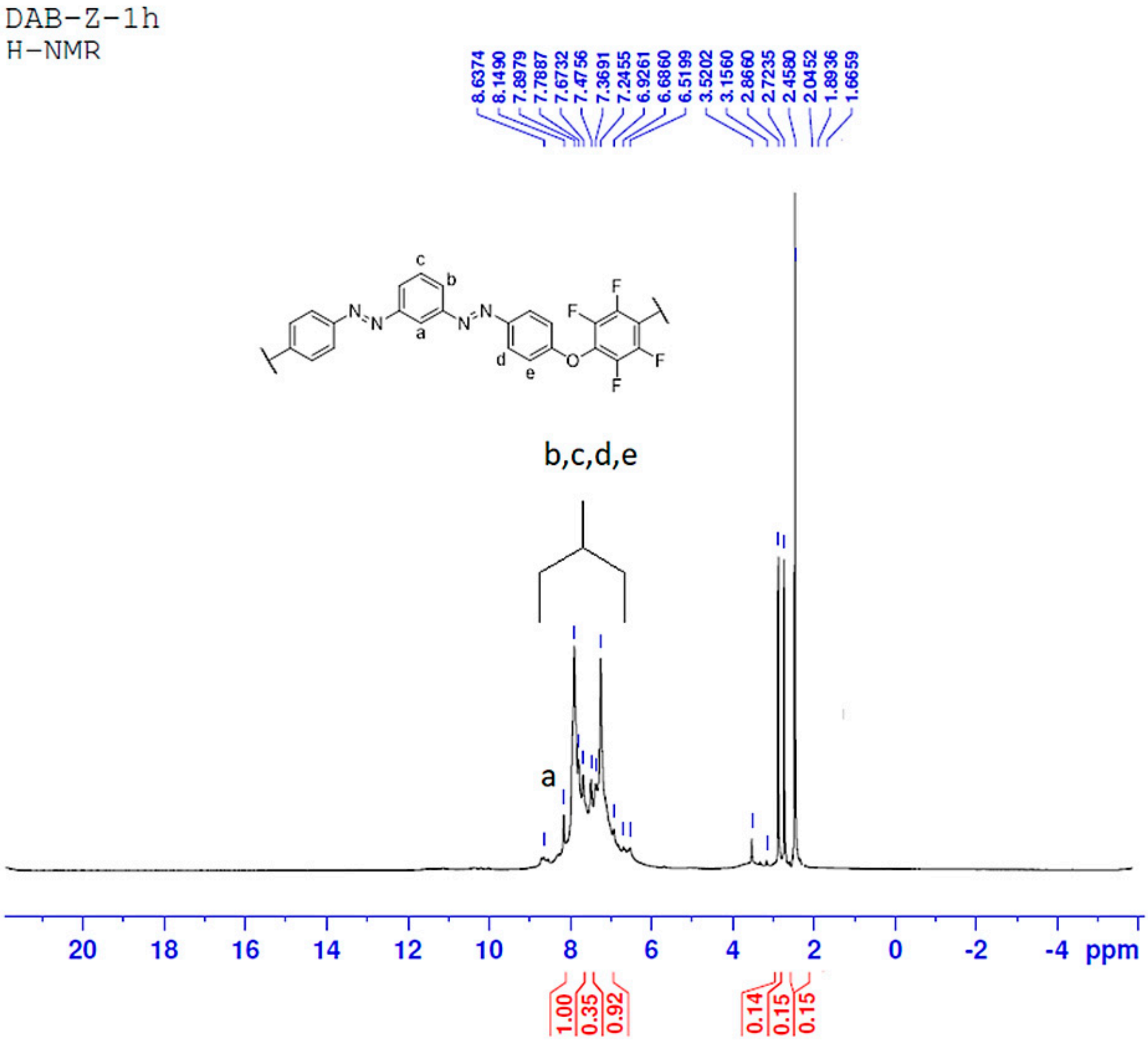

Figure S9. ^{1}H NMR spectra of DAB-Z-1h.

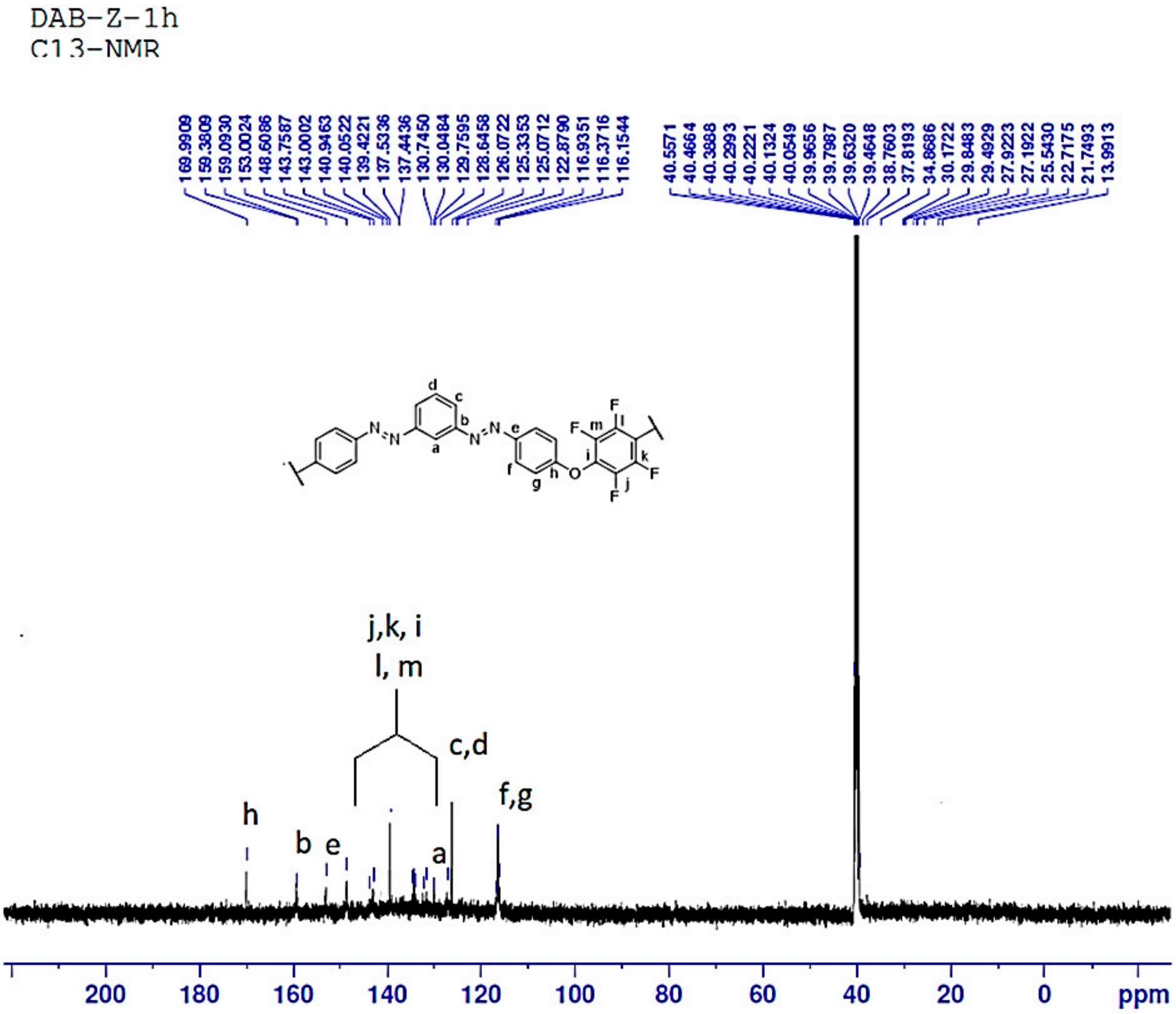

Figure S10. ^{13}C NMR spectra of DAB-Z-1h.

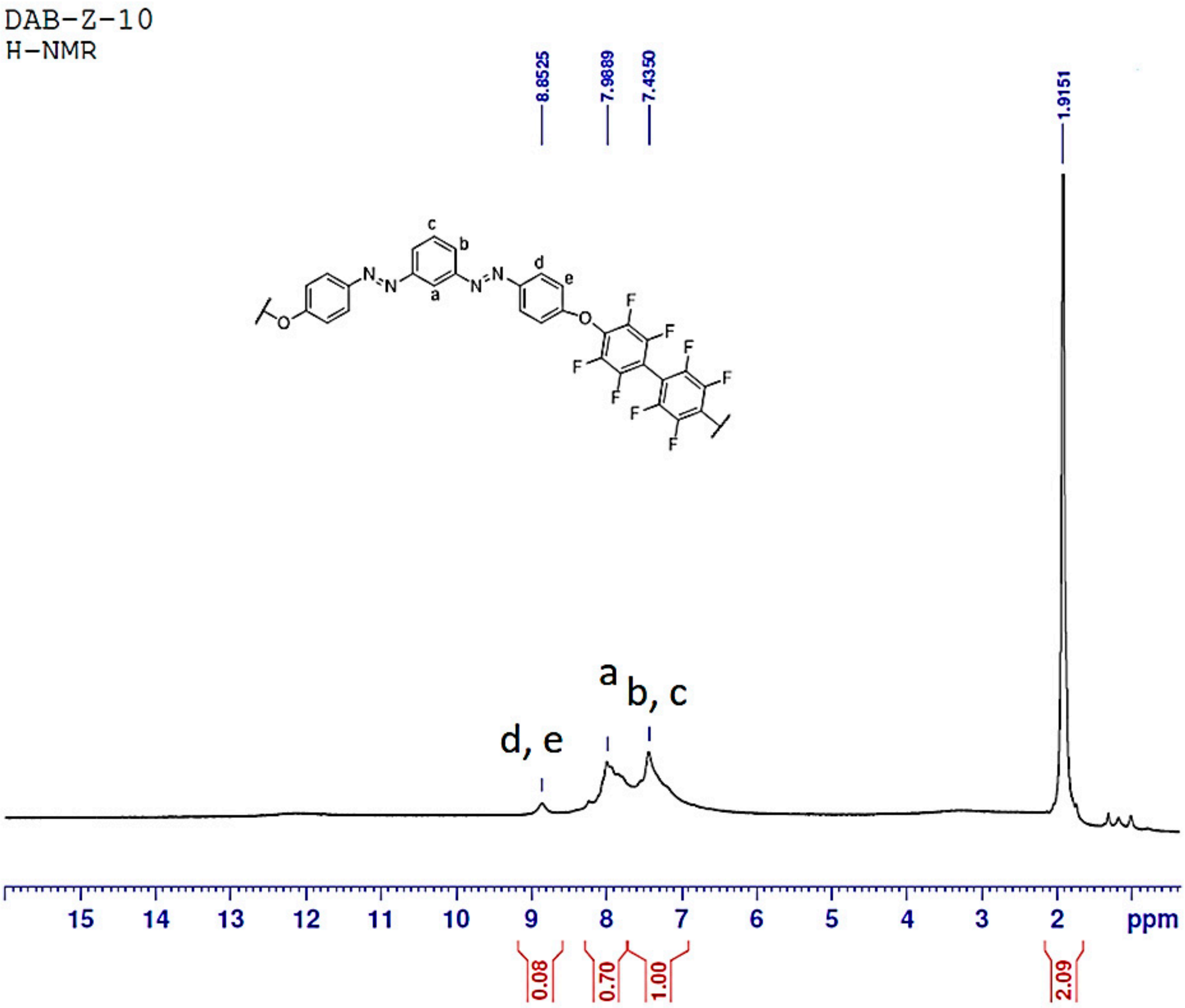

Figure S11. ^{1}H NMR spectra of DAB-Z-1O.

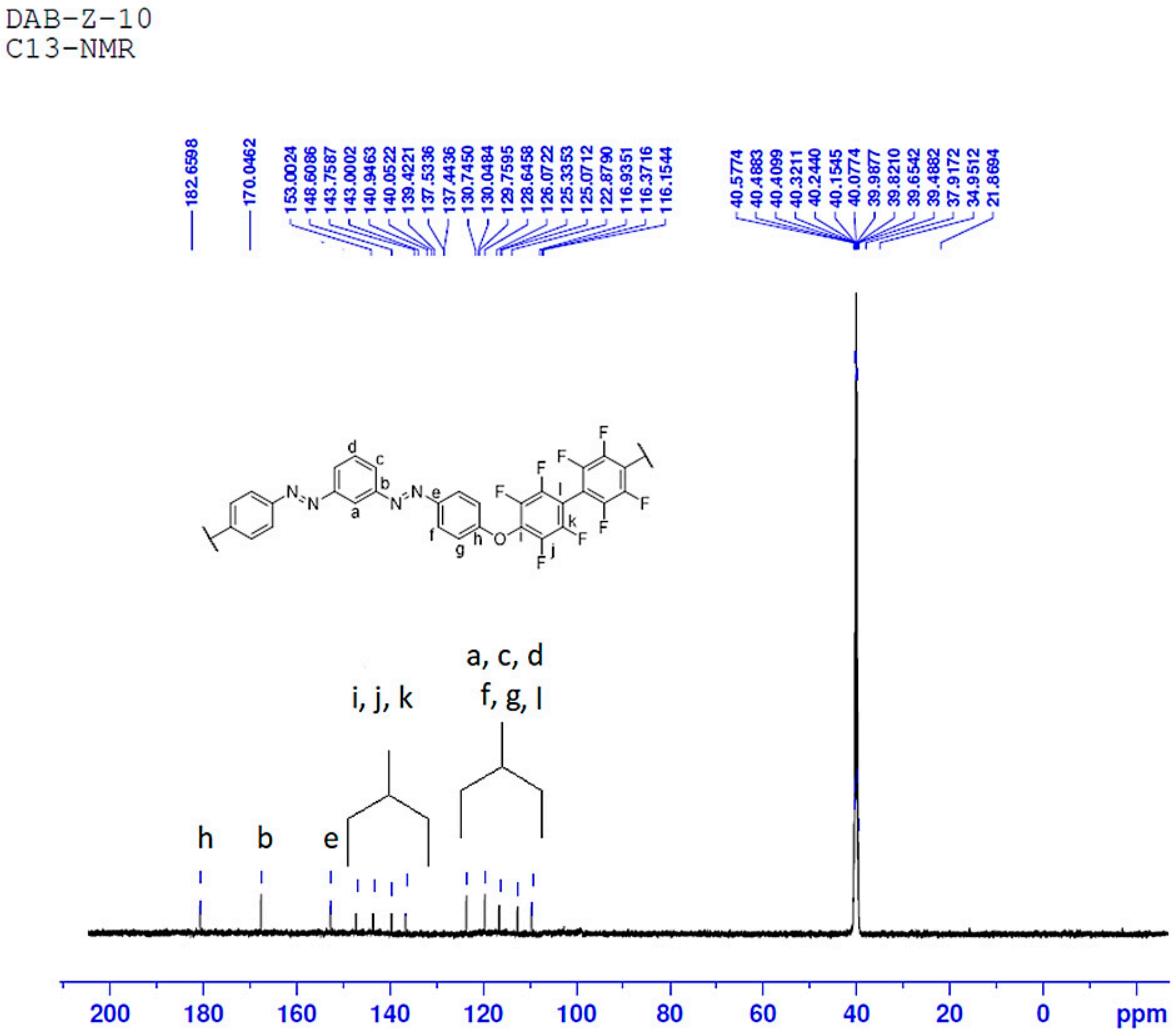

Figure S12. ^{13}C NMR spectra of DAB-Z-1O.

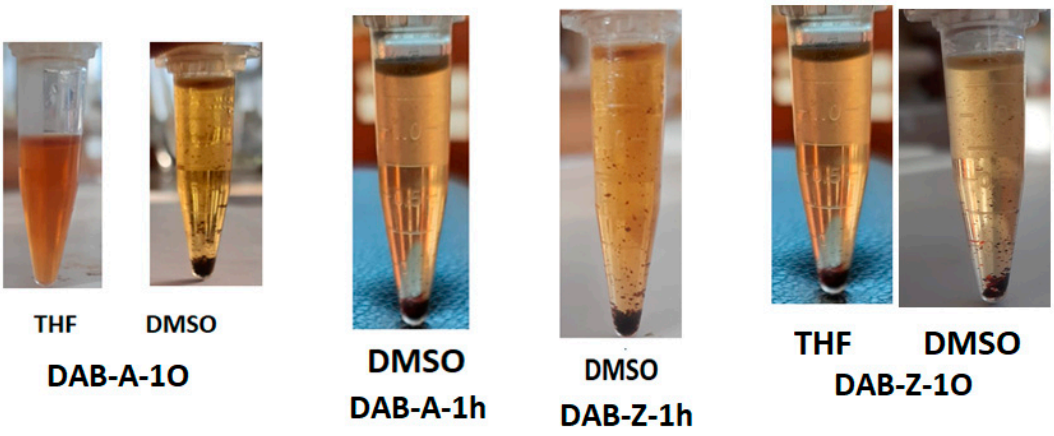

Figure S13. Solubility behavior of the polymers in THF and DMSO. (Note: DAB-A-1h, DAB-Z-1h were completely insoluble in THF).

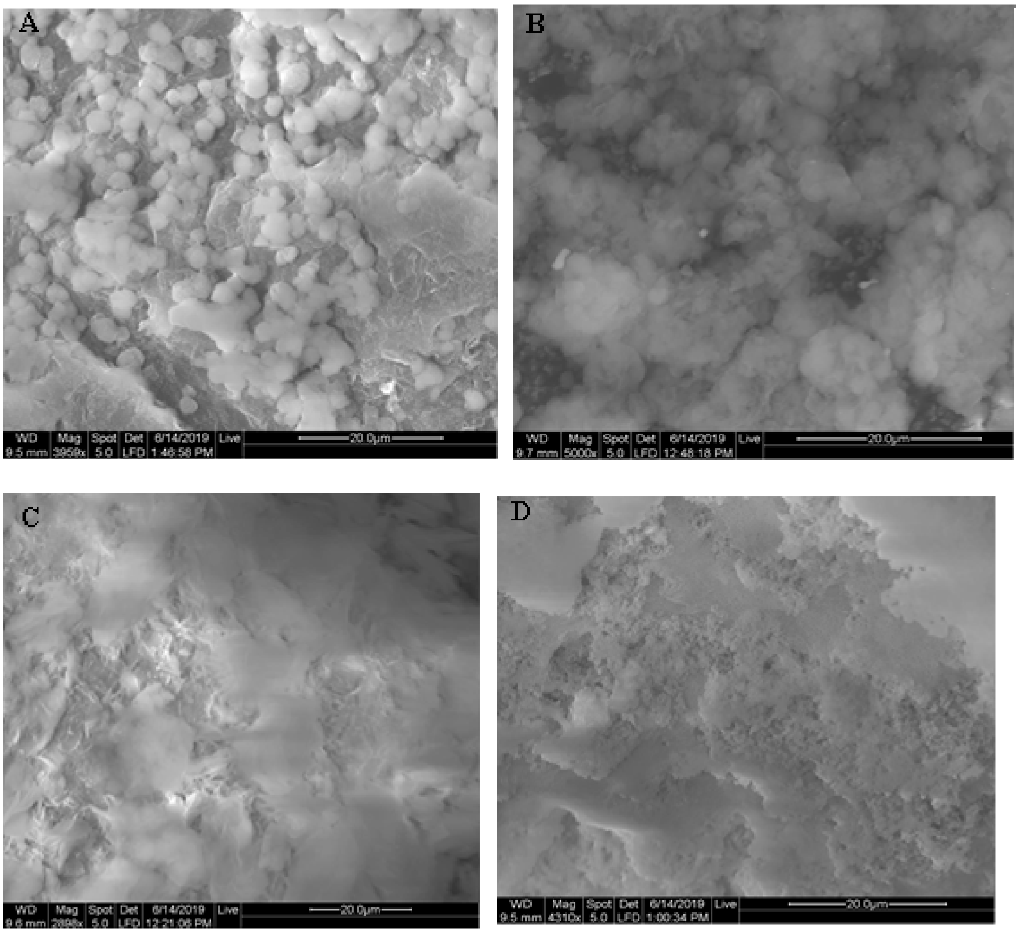

Figure S14. The SEM of polymers: (A) DAB-Z-1O, (B)DAB-Z-1h (C) DAB-A-1O. and (D) DAB-A-1h.

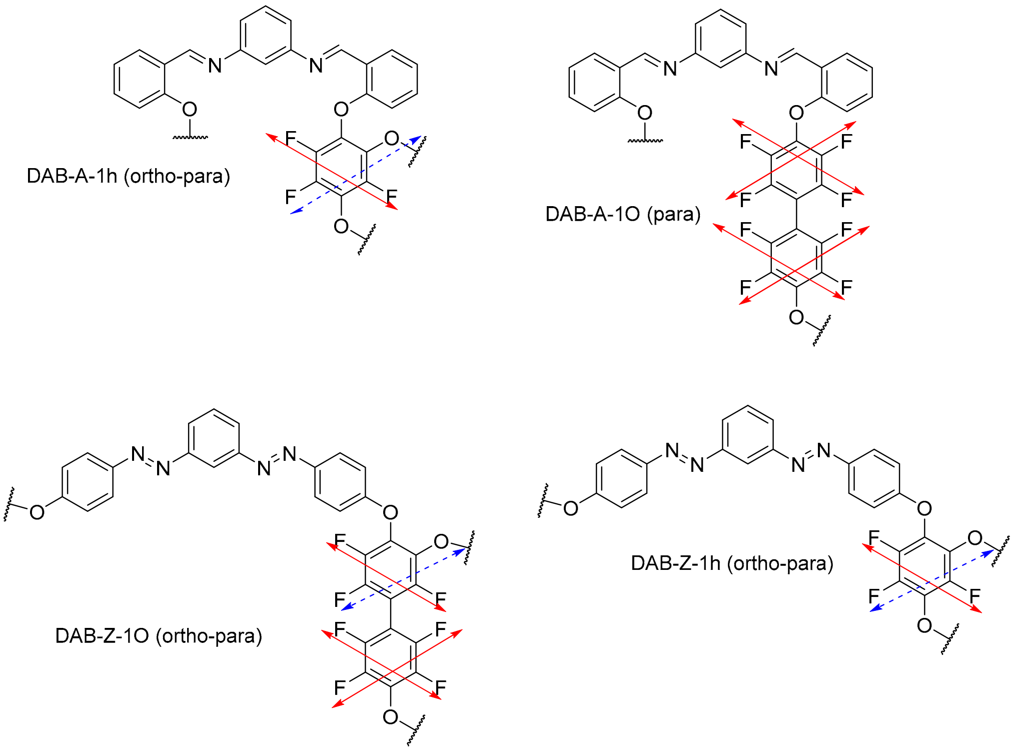

Figure S15. Proposed sites of zero and non-zero dipole moments of the polymers; (bold arrow create μ = 0, dashed arrow creates μ > 0).

Part S2: BET surface area calculations

The BET surface area of the polymers was determined by using Multi-point BET plot by using eq (1), where Xm is the monolayer capacity, P/P0 relative pressure, and C is constant. The plot of $\frac{1}{X\left[\left(\frac{p0}{p}\right)-1\right]}$ Vs $\left(\frac{P}{P0}\right)$ is performed by using, at minimum, three data points, in the P/P0 range 0.025 to 0.30, which should give a linear relation with a positive slope. It is well-known that at relative pressures higher than 0.5, there is the onset of capillary condensation, and at relatively low pressures only a monolayer gas adsorption occurs. gives liners plot with positive slope.

$$\frac{\mathbf{1}}{\boldsymbol{X}\left[\left(\frac{\boldsymbol{p0}}{\boldsymbol{p}}\right)-\mathbf{1}\right]}=\frac{\mathbf{1}}{\boldsymbol{XmC}}+\left(\frac{\boldsymbol{C}-\mathbf{1}}{\boldsymbol{XmC}}\right)*\left(\frac{\boldsymbol{P}}{\boldsymbol{P0}}\right) \quad (1)$$

When the values of the slope and y-intercept is determined by using least squares regression, the monolayer capacity Xm can be calculated by applying Equation (2).

$$\boldsymbol{Xm}=\left(\frac{\mathbf{1}}{\boldsymbol{slope}\ (\boldsymbol{s})+\boldsymbol{intercept}\ (\boldsymbol{i})}\right) \quad (2)$$

Once **Xm** is determined, the total surface area **SA** can be calculated from eq (3), where **Av** is Avogadro's number (6.022 *10^{23}), **Am** is the cross sectional area of the adsorbate and equals 0.138 nm^2 for an absorbed argon molecule, and **Mv** is the molar volume, which equals 22414 mL as STP conditions.

$$\boldsymbol{SA}\ \left(\frac{\boldsymbol{m^2}}{\boldsymbol{g}}\right)=\left[\frac{\boldsymbol{Xm}\frac{\boldsymbol{cc}}{\boldsymbol{g}}*\boldsymbol{Am}\ (\boldsymbol{nm^2})*\boldsymbol{Av}}{\mathbf{22414}\ \boldsymbol{cc}}\right] \quad (3)$$

According to the above equations, the calculated Xm and SA values along with the slope, and intercepts of the plots are summarized in table S3 [2-4].

Table S3. BET plots data of the polymers.

**Equation**	**y = a + b*x**			
**Plot**	DAB-A-1O	DAB-A-1h	DAB-Z-1h	DAB-Z-1O
**Weight**	No Weighting			
**Intercept (i)**	-4.58E-09	-2.795E-09	-2.677E-09	-1.678E-09
**Slope (s)**	0.0129972	0.0091831	0.0075039	0.0048092
**Xm=1/(s+i)**	76.93967565	108.895722	133.2640836	207.9348642
**SA (surface area)**	285.2665312	403.7488409	494.0985588	770.9527876
**Residual Sum of Squares**	3.61E-05	1.99E-05	1.12E-05	4.99E-06
**Pearson's r**	0.994560004	0.99375191	0.994836658	0.994574501
**R-Square (COD)**	0.989149602	0.987542859	0.989699976	0.989178439
**Adj. R-Square**	0.986437003	0.984428574	0.98712497	0.986473049

References

1. Kaseman, D. C.; Janicke, M. T.; Frankle, R. K.; Nelson, T.; Angles-Tamayo, G.; Batrice, R. J.; Magnelind, P. E.; Espy, M. A.; and Williams, R. F. Chemical Analysis of Fluorobenzenes via Multinuclear Detection in the Strong Heteronuclear J-Coupling Regime. *Appl. Sci* **2020**, 10, 3836
2. Bae, Y.-S.; Yazaydın, A.Ö. ; and Snurr, R.Q. Evaluation of the BET Method for Determining Surface Areas of MOFs and Zeolites that Contain Ultra-Micropores. *Langmuir* **2010**, 26, 5475-5483.
3. Merukan Chola, N.; Gajera, P.; Kulkarni, H.; Kumar, G.; Parmar, R.; Nagarale, R. K.; and Sethia, G. Sorption of Carbon Dioxide and Nitrogen on Porous Hyper-Cross-Linked Aromatic Polymers: Effect of Textural Properties, Composition, and Electrostatic Interactions. ACS Omega **2023**, 8, 28, 24761–24772
4. Shi, K.; Li, Z.; Anstine, D. M.; Tang, D.;Colina, C. M.; Sholl, D. S.; Siepmann, J. I.; and Snurr, R. Q. Two-Dimensional Energy Histograms as Features for Machine Learning to Predict Adsorption in Diverse Nanoporous Materials. *J. Chem. Theory Comput* **2023**, 19, 14, 4568–4583.
